# Metabolic and transcriptional alternations for defense by interfering *OsWRKY62* and *OsWRKY76* transcriptions in rice

**DOI:** 10.1038/s41598-017-02643-x

**Published:** 2017-05-30

**Authors:** Xiaoxing Liang, Xujun Chen, Cheng Li, Jun Fan, Zejian Guo

**Affiliations:** Key Laboratory of Pest Monitoring and Green Management, MOA, Department of Plant Pathology, China Agricultural University, Beijing, 100193 China

## Abstract

Metabolomic and transcriptomic approaches were used to dissect the enhanced disease resistance in the plants harbouring a RNA interfering construct of *OsWRKY62* and *OsWRKY76* (dsOW62/76) genes. The primary metabolic pathways were activated in dsOW62/76 compared with wild-type (ZH17) plants, revealed by increased accumulation of amino acids and constituents of citric acid cycle *etc*. Contents of phenolic acids derived from phenylpropanoid pathway were elevated in dsOW62/76 plants. Importantly, phenolamides, conjugates of the phenolic acids with amines, were detected in large number and mostly at higher levels in dsOW62/76 compared with ZH17 plants; however, the free pools of flavonoids were mostly decreased in dsOW62/76. Salicylic acid (SA) and jasmonic acid (JA)/JA-Ile contents were increased in dsOW62/76 and knockout lines of individual *OsWRKY62* and *OsWRKY76* genes. Transcription of isochorismate synthase (*OsICS1*) gene was suppressed in dsOW62/76 and in MeJA-treated rice plants, whereas the transcription level of cinnamoyl-CoA hydratase-dehydrogenase (*OsCHD*) gene for β-oxidation in peroxisome was increased. The calli with *OsCHD* mutation showed markedly decreased SA accumulation. These results indicate that OsWRKY62 and OsWRKY76 function as negative regulators of biosynthetic defense-related metabolites and provide evidence for an important role of phenylpropanoid pathway in SA production in rice.

## Introduction

Plants must arise proper responses to overcome the adverse environmental stimuli such as drought stress, pathogen infection, and herbivore attack. Among the external stresses, plant disease caused by pathogen infection is one of the severe obstacles for crop production. The difficulties of disease resistance breeding are (i) frequent breakdown of resistance (*R*) gene mediated defenses; (ii) fitness cost of activation of defense responses. It is therefore important to understand the molecular processes related to plant defenses and provide useful genes for molecular breeding of durable disease resistance.

Plants constitutively produce numerous metabolites which may form the chemical barriers that prevent pathogen invasion (constitutive resistance). When the constitutive resistance fails to prevent entry of pathogens, plants are able to activate series of defense responses including the formation of secondary metabolites, such as phenolics, terpenoids, glucosinolates and phytoalexins. Many of these metabolites are proven or potential antimicrobial active. For example, the soluble phenylpropanoids, such as sinapoyl glucose, coniferyl alcohol, and coniferin, are inhibitors of fungal growth and important for the defense of *Arabidopsis thaliana* against *Verticillium longisporum* ^1^. Moreover, some metabolites have been characterized as inducers of defense or as priming agents that are able to potentiate host responses. Phytohormone salicylic acid (SA) has been reported to play an important role in defense priming^2, 3^. Hexanoic acid has been shown as a broad-spectrum natural inducer that protects tomato and Arabidopsis plants against *Botrytis cinerea* ^4–6^. Pipecolic acid (Pip), the lysine degradation product, has been found to be essential to systemic acquired resistance and priming in Arabidopsis plants^7^. Azelaic acid, a catabolite of free unsaturated acids, primes the plant to accumulate higher SA levels upon bacterial infection^8^. Recently, the resistance-associated 1-methyltryptophan has been demonstrated to protect tomato against *Pseudomonas syringae* pv. *tomato* (*Pst*) DC3000 and *B. cinerea* ^9^. With continuing identification of natural priming activators and dissection of their molecular mechanisms, it is expected that the priming compounds and/or their derivatives will increasingly be put into practice.

Transcriptomic and metabolomic studies have provided insights into signaling networks associated with biotic and abiotic stresses. Maruyama *et al*. performed an integrated analysis of the metabolites and transcriptomic profiling in rice plants subjected to cold and dehydration treatments and found that the stresses increase abscisic acid (ABA) signaling and decrease cytokinin signaling^10^. In response to UV-B treatment, Arabidopsis undergoes extensive reprogramming in both primary and specialized metabolism^11^. Rapid alterations in levels of phenolics, amino acids and molecules associated with amelioration of redox stress were reported during infection of *A. thaliana* with virulent *Pst* DC3000^12^. Identical patterns of metabolic changes were found during *Magnaporthe oryzae* infections in barley, rice and *Brachypodium distachyon* plants using targeted metabolite profiling by GC-MS^13^. The accumulation of malate, polyamines, lignin precursors and compounds that influence the prevalence of reactive oxygen species (ROS) was observed in the pre-symptomatic tissues. The fungal pathogen deploys a common metabolic reprogramming strategy in diverse host species to establish a nutritional interface at early stage of infection^13^. Thus, omic studies are able to reveal the significance of metabolites in plant adaptive responses to environmental stresses.

Transcription factors (TFs) of MYBs and WRKYs are important regulators of biosynthetic metabolites^14, 15^. GaWRKY1 is a positive regulator of the sesquiterpene synthase gene leading to the production of phytoalexin gossypol in cotton^16^. CrWRKY1 from *Catharanthus roseus* was found to directly regulate the transcription of the gene encoding tryptophan decarboxylase (TDC), the enzyme catalyzing the formation of indolic tryptamine^17^. AtMYB12 has been characterized as a specific transcription factor capable of regulating flavonol biosynthesis in Arabidopsis^18, 19^ as well as in tomato and tobacco^20, 21^. *Nicotiana attenuata* NaMYB8 regulates the accumulation of phenolamides, which may be essential for defense against chewing herbivores^22^. Three acyltransferase genes have been characterized to be targets of NaMYB8 and are responsible for phenolamide biosynthesis in *N. attenuata* plants^23^. However, much more needs to be explored regarding genes regulating the biosynthesis of metabolites, which is also highly useful to metabolic engineering of crops to produce useful chemicals.

We have shown in an earlier study that OsWRKY62 and OsWRKY76 are repressors of defense mechanisms in rice plants. Plants harboring the construct of knock-down both *OsWRKY62* and *OsWRKY76* (dsOW62/76) show enhanced accumulation of abnormal transcripts of both genes in particular, leading to lesion mimic phenotype and immunity to pathogens of *M. oryzae* and *Xanthomonas oryzae* pv. *oryzae* (*Xoo*)^24^. To explore the underlying mechanisms at metabolic level, we performed a comprehensive profiling study of metabolites, phytohormones and phytoalexins in dsOW62/76 in comparison with the wild-type (ZH17) plants. In parallel, we analyzed the transcriptomics of dsOW62/76 and ZH17 plants, and in particular, the genes related to the biosynthetic pathways of various metabolites. These results indicate that OsWRKY62 and OsWRKY76 play important roles in regulation of biosynthetic defense-related metabolites.

## Results

### Metabolomic and transcriptomic analyses of dsOW62/76 and ZH17 plants

Knockout lines of individual *OsWRKY62* (W62-KO) and *OsWRKY76* (W76-KO) gene and the knockdown line of both genes (dsOW62/76) were initially analysed for metabolomic changes compared with the control (ZH17) plants (Supplementary Fig. S1A). Most significant changes in numbers and levels of metabolites were observed between dsOW62/76 and ZH17 plants (Supplementary Fig. S1B,C), implying that previously observed abnormal accumulation of *OsWRKY62.2* and *OsWRKY76.2* transcripts in dsOW62/76 plants^24^ might be involved in the massive change in the metabolic profiles. Therefore, we mainly investigated the change of 611 metabolites from rice plants (Supplementary Table S1) in the dsOW62/76 line for further studies.

In addition, we also performed transcriptomic analysis and found prominent upregulation of gene transcriptions in dsOW62/76 line compared with that of ZH17 plants (Supplementary Table S2). Some of the genes involved in the analyzed metabolite biosynthetic pathways were identified and shown accordingly in the figures.

### Variation of primary metabolomic in dsOW62/76 plants

**Figure 1:**
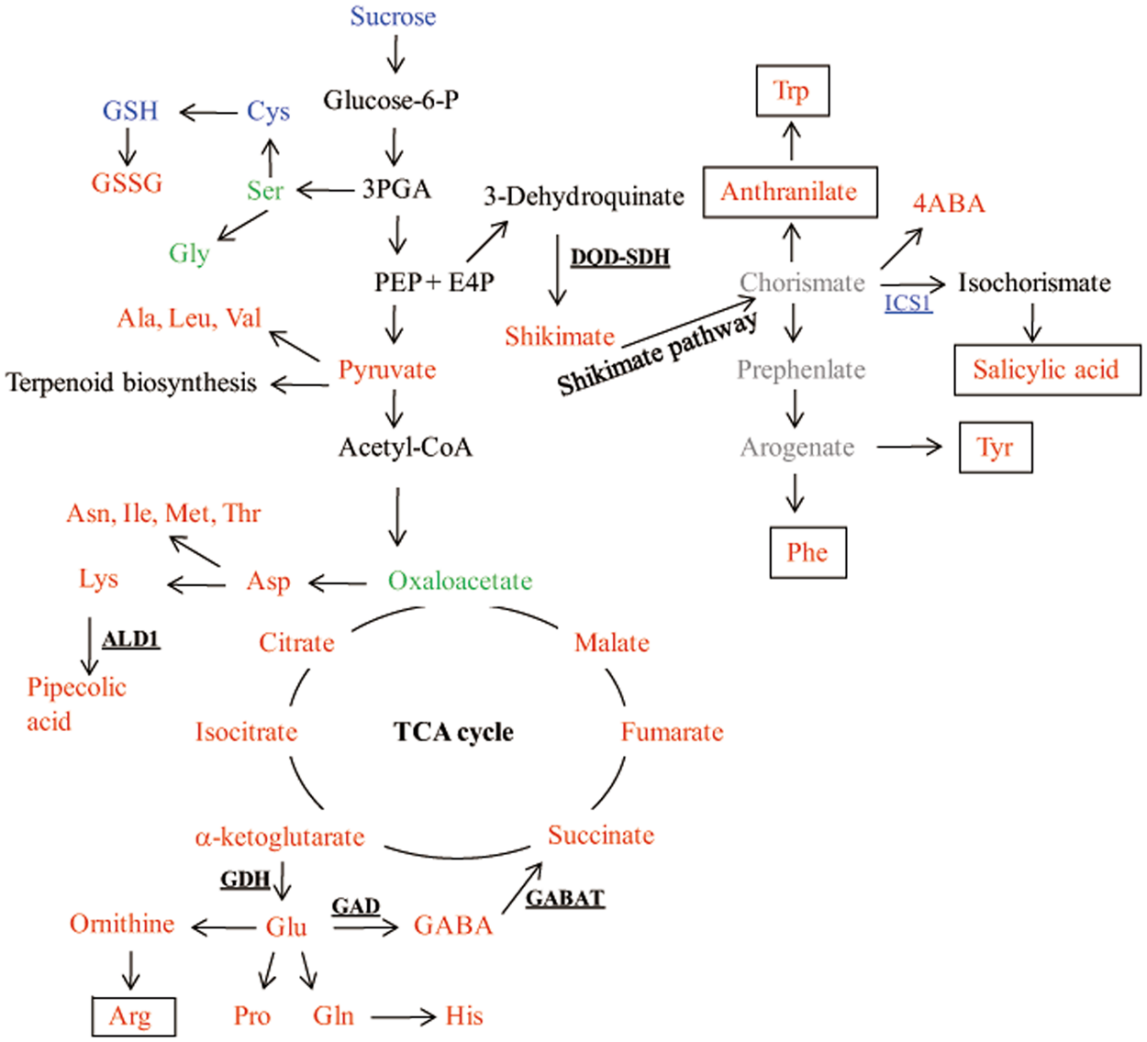
Schematic overview of the changes in the primary metabolic biosynthesis pathways compared between dsOW62/76 and ZH17 plants. The compound in red, blue, and green indicated that its accumulation was increased, decreased, and not changed significantly in dsOW62/76 in related to ZH17 plants. The compound undetectable was in gray. Genes or enzymes were shown as underlined. The genes analyzed by qPCR were in red or blue, representing increase or decrease their transcription levels in dsOW62/76. Some of the genes upregulated in the transcriptome analysis were shown in bold. The compounds boxed are precursors may be mentioned in the later studies. Abbreviations: 4ABA, 4-aminobenzoic acid; E4P, erythrose 4-phosphate; GABA, γ-aminobutyric acid; GSH, glutathione; GSSG, glutathione oxidized; PEP, phospho*enol*pyruvate; 3PGA, 3-phosphoglycerate; ALD1, aminotransferase 1; DQD-SDH, 3-dehydroquinate dehydratase-shikimate dehydrogenase; GABAT, 4-aminobutyrate-pyruvate transaminase; GAD, Glu decarboxylase; GAH, Glu dehydrogenase; ICS, isochorismate synthase.

The tricarboxylic acid (TCA) pathway in dsOW62/76 plants was up-regulated compared to ZH17 controls, which was indicated by the accumulation of multiple intermediates of the pathway including citrate, 2-ketoglutarate (2-OG), fumarate, malate, and succinate (Fig. 1; Supplementary Table S3). It has been shown that intermediates of the TCA cycle within the mitochondrial matrix may be used elsewhere in many other fundamental biosynthetic processes of the cell^25, 26^.

Biosynthetic pathways of Asp and Glu initiate from oxaloacetate and 2-OG, respectively. Levels of Asp family amino acids (Asn, Met, Ile, Thr, and Lys) were increased in dsOW62/76 plants. The accumulation of Lys-derived Pip, a priming agent, was enhanced 9.4-fold in dsOW62/76, which was consistent with the transcriptional upregulation of *OsALD1* gene (Fig. 1; Supplementary Table S4), the ortholog of *ALD1* required for Pip biosynthesis in Arabidopsis^8^. Likewise, the accumulation of Glu and its family amino acids (Gln, His, Pro, Arg and ornithine (Orn)) was also increased (Fig. 1). Glu is a precursor used by Glu decarboxylase (GAD) to catalyze the formation of γ-aminobutyric acid (GABA), an important defense- and stress-related metabolite, which was observed to accumulate strikingly in dsOW62/76 plants (Supplementary Table S3). The activation of GABA shunt in dsOW62/76 plants was further corroborated by data showing the induction of genes encoding enzymes involved in the biosynthetic route including GAD, Glu dehydrogenase (GDH), and 4-aminobutyrate-pyruvate transaminase (GABAT) (Supplementary Table S4).

Ser and its family amino acids (Cys and Gly) are converted from 3-phosphoglycerate (3PGA). Cys leads to form glutathione (GSH), which is a component of ascorbate-GSH cycle considered to play a crucial role in adjusting the cellular ROS level. Since dsOW62/76 plants showed spontaneous cell death^24^, cellular redox homeostasis would be retrimmed. As expected, the level of the oxidized GSH (GSSG) increased in dsOW62/76 with decrease of GSH and its precursor Cys along with ascorbate and dehydroascorbate (Fig. 1; Supplementary Table S3), indicating that the plants were under oxidative stress. GSH is also required by GSH *S*-transferase (GST) and glutaredoxin to reduce their targets. Upregulation of glutaredoxin and *GST* genes in dsOW62/76 plants implied elevated requirement of GSH to maintain cellular redox status (Supplementary Table S4).

Phopho*enol*pyruvate (PEP) and erythrose 4-phosphate (E4P) are intermediates of the pentose phosphate and glycolytic pathways, respectively, and are converted in the shikimate pathway to form the chorismate, an important precursor for most benzenoid compounds including aromatic amino acids^27^. In dsOW62/76 plants, we observed differential upregulation of genes encoding enzymes for chorismate biosynthesis including fused 3-dehydroquinate dehydratase-shikimate dehydrogenase (*DQD-SDH*), shikimate kinase (*SK*), and 5-*enol*pyruvylshikimate and 3-phosphate synthase (*EPSPS*) (Supplementary Table S4). Consequently, Phe, Tyr and Trp were found to accumulate to higher levels in dsOW62/76 than in ZH17 plants (Supplementary Table S3). Increases of most of the amino acids detected implied large scale activation of metabolomics in dsOW62/76 since some of these amino acids are essential precursors for the biosynthesis of many primary and/or secondary metabolites.

### Upregulation of phenylpropanoid biosynthetic pathway in dsOW62/76 plants

**Figure 2:**
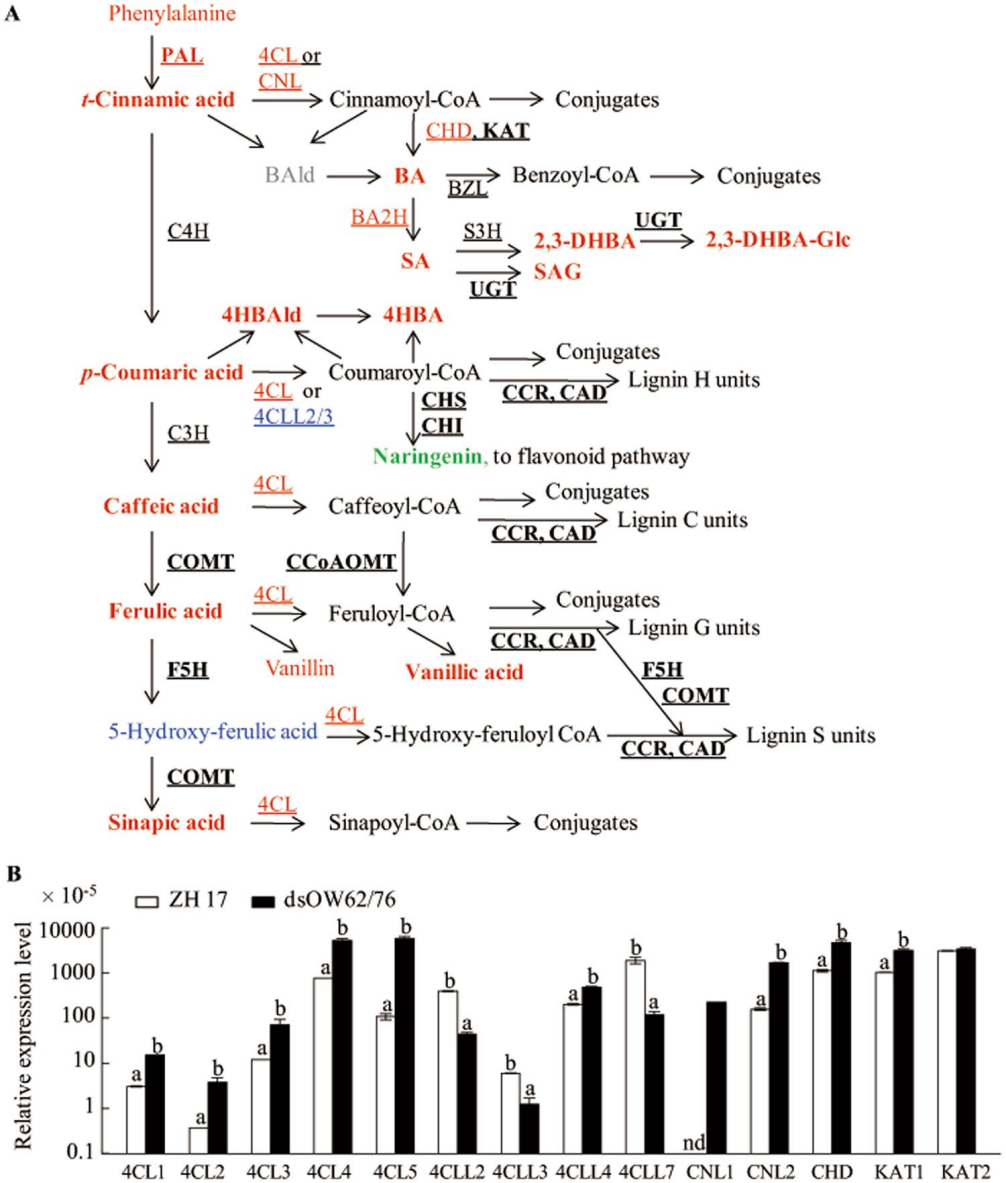
Schematic representation of variations in the phenylpropanoid pathway (**A**) and gene expressions (**B**). Free phenolic acids can be activated by ligases (4CLs) to form phenoloyl-CoAs, which may conjugate with amines to produce phenolamides or used as precursors for lignan biosynthesis. Feruloyl-CoA can be converted to coniferaldehyde by caffeic acid 3-*O*-methyltransferase (CCR) and further to coniferyl alcohol by cinnamyl alcohol dehydrogenase (CAD). Ferulate 5-hydroxylase (F5H) can catalyze the 5-hydroxylation of coniferaldehyde and coniferyl alcohol and then the products are methylated by caffeic acid/5-hydroxyferulic acid *O*-methyltransferase (COMT) to produce sinapaldehyde and sinapyl alcohol. Phenoloyl-CoAs generated by ligase like enzymes (4CLLs, or CNLs) in peroxisome may go through β-oxidative pathway to form benzenoid compounds. The font colors and shapes represent the same meanings as described in Fig. 1. The chemicals in bold were the compounds labeled by isotopes in the [^2^H_8_]Phe feeding experiments. A rice *ubiquitin* gene was used as the internal standard for qPCR analysis. Experiments were performed twice with similar results. Values marked with different letters indicate statistically significant differences as analyzed by SAS software (Duncan’s multiple range test, α = 0.05). nd for undetectable. Abbreviations: BA, benzoic acid; BAld, benzaldehyde; 2,3-DHBA, 2,3-dihydroxy BA; 2,3-DHBA-Glc, 2,3-DHBA glucoside; 4HBA, 4-hydroxy benzoic acid; 4HBAld, 4-hydroxy benzaldehyde; SA, salicylic acid; SAG, SA glucoside; BA2H, BA 2-hydroxylase; BZL, benzoyl-CoA:ligase; C3H, *p*-coumarate 3-hydroxylase; C4H, cinnamate 4-hydroxylase; CCoAOMT, caffeoyl-CoA 3-*O*-methyltransferase; 4CL, 4-coumaroyl-CoA:ligase; 4CLL, 4CL like; CNL, cinnamoyl-CoA:ligase; CHD, cinnamoyl-CoA hydratase-dehydrogenase; CHI, chalcone isomerase; CHS, chalcone synthase; KAT, 3-ketoacyl-CoA thiolase; PAL, phenylalanine ammonia-lyase; S3H, SA 3-hydroxylase; UGT, UDP-glycosyltransferase.

The general phenylpropanoid pathway is associated with the shikimate route in plants. Phenylalanine ammonia-lyase (PAL) catalyzes the elimination of ammonia from Phe to produce *trans*-cinnamic acid (*t*-CA), which is subsequently converted to *p*-coumaric, caffeic, ferulic and sinapic acids. Accumulation of these compounds (Fig. 2A; Supplementary Table S5) and upregulation of some of the genes involved in the phenylpropanoid pathway (Supplementary Table S6) were found in dsOW62/76 plants. The phenolic acids are catalyzed by 4-coumarate:CoA ligase (4CL) or cinnamate:CoA ligase (CNL) into acyl CoA esters, which may further conjugate with amines to form phenolamides and/or be used as precursors for lignin biosynthesis. Five 4CLs and two CNLs have been reported in rice^28, 29^. Transcript levels of these *CNL* and *4CL* genes strongly increased in dsOW62/76 plants (Fig. 2B), suggesting that the enzymes potentially participated in the generation of CoA esters.

The accumulation of benzenoid compounds in dsOW62/76 plants was shown in Supplementary Table S5, including *p*-aminobenzoic acid (4ABA), BA, *p*-hydroxybenzaldehyde (4HBAld), *p*-hydroxybenzoic acid (4HBA), vanillin and vanillic acid (VA). In plants, benzenoid products can be synthesized from the intermediates of shikimate pathway or chorismate in plastid, and they also can be derived from Phe via *t*-CA to shorten the C_3_ side chain by two carbon units in peroxisome^27, 30^. The first step for β-oxidation is the acyl-activation of phenolic acids by 4CLs in the peroxisome. Transcription of rice 4CL like genes such as *4CLL2* and *4CLL3*, close homologs of *At4g19010*, was reduced in dsOW62/76, while the transcriptional level of rice *4CLL4*, orthologous to *At1g20510*, were increased (Fig. 2B; Supplementary Fig. S2). The peroxisome localized acyl-activating enzymes of At4g19010 and At1g20510 are required for ubiquinone and jasmonate biosynthesis in Arabidopsis, respectively^31, 32^. The data implied the existence of potential differences in the requirement of ubiquinone and jasmonate between dsOW62/76 and ZH17 plants.

To efficiently annotate the metabolites of the phenylpropanoid pathway, we used [^2^H_8_]Phe to track the biosynthetic flux. The isotope labeled compounds are shown in Fig. 2A and Table 1 and their MS/MS ions are shown in Supplementary Table S7. Samples were collected at 12 h (early) and 48 h (late) post feeding treatment of dsOW62/76 and ZH17 plants with [^2^H_8_]Phe. The detection of deuterium labeled SA and its derivatives such as SA glucoside (SAG) and 2,3-dihydroxybenzoic acid (2,3-DHBA) confirmed that phenylpropanoid pathway mediates the biosynthesis of SA via BA^33^ and indicated the existence of multiple ways to maintain SA homeostasis. In petunia, PhCHD (cinnamoyl-CoA hydratase-dehydrogenase) is characterized to efficiently convert cinnamoyl-CoA (Cin-CoA) to 3-oxo-3-phenylpropionoyl-CoA (3O3PP-CoA), which is the substrate for the final step in the BA β-oxidation pathway^30^. Consistent with these observations, qRT-PCR results showed that transcription of *LOC_Os02g17390* (*OsCHD*) gene, the closest homolog of *PhCHD* (Supplementary Fig. S2), was upregulated in dsOW62/76 plants (Fig. 2B). Also, *OsKAT1* (3-ketoacyl-CoA thiolase 1) homologous to *PhKAT*, catalyzing the formation of benzoyl-CoA (Ben-CoA) from 3O3PP-CoA, was strongly induced in dsOW62/76 (Fig. 2B; Supplementary Fig. S2; Supplementary Table S6). These data may reflect a higher rate of biosynthesis SA from *t*-CA in dsOW62/76 than ZH17 plants as observed in the isotope chasing experiments.

**Table 1:** Analysis of the compounds labeled by ^2^H isotope in rice leaves fed with [^2^H_8_]Phe for 12 h.

Compounds	ZH17		dsOW62/76	
	H	D	H	D
Phenylalanine	128.9 ± 15.3	164.5 ± 20.1	118.1 ± 3.8	198.6 ± 5.2
Benzoic acid	53.83 ± 13.56	1.41 ± 0.32	99.56 ± 3.67	3.39 ± 0.11
*t*-Cinnamic acid	0.03 ± 0.03	0.01 ± 0.01	0.16 ± 0.01	0.06 ± 0.01
2,3-DHBA	3.1 ± 0.75	0.24 ± 0.07	11.96 ± 0.07	1.15 ± 0.01
2,3-DHBA-Glc	85.49 ± 0.39	4.88 ± 0.14	361.7 ± 3.4	20.49 ± 0.08
4-Hydroxybenzaldehyde	0.24 ± 0.06	nd	0.41 ± 0.08	nd
4-Hydroxy benzoic acid	3.53 ± 0.34	nd	10 ± 0.35	0.49 ± 0.02
2-Hydroxycinnamic acid	65 ± 7.92	0.71 ± 0.16	75.24 ± 2.8	1.38 ± 0.04
4-Hydroxycinnamic acid	0.32 ± 0.2	0.02 ± 0.02	0.74 ± 0.03	0.09 ± 0.01
3H2HPPA	0.21 ± 0.02	0.15 ± 0.05	0.31 ± 0.01	0.47 ± 0.01
3H4HPPA	0.1 ± 0.02	0.13 ± 0.04	0.28 ± 0.03	0.23 ± 0.02
3H3Me4HPPA	0.1 ± 0.02	nd	0.2 ± 0.01	nd
3HPPA	0.12 ± 0.05	nd	0.1 ± 0.01	0.12 ± 0.02
SAG	32.64 ± 1.41	0.19 ± 0.01	104.4 ± 3.05	3.99 ± 0.11
Salicylic acid	208.08 ± 29.41	1.78 ± 0.16	455.48 ± 2.33	21.01 ± 0.92
Vanillic acid	0.19 ± 0.06	0.09 ± 0.02	0.39 ± 0.12	0.17 ± 0.01
Vanillin	0.13 ± 0.04	nd	0.18 ± 0.01	nd

nd, not detected; **D** for deuterium labeled and **H** for non isotope labeled compounds; The internal standard was [^2^H_6_]ABA (10 ng). Data are means ± SE of three replicates. The experiments were repeated three times with similar results. The metabolite contents presented were normalized ng mg^−1^ DW. Abbreviations: 2,3-DHBA, 2,3-dihydroxylbenzoic acid; 2,3-DHBA-Glc, 2,3-DHBA glucoside; 3H2HPPA, 3-hydroxy-(2-hydroxyphenyl) propionic acid; 3H4HPPA, 3-hydroxy-(4-hydroxyphenyl) propionic acid; 3H3Me4HPPA, 3-hydroxy- (3-methoxy-4-hydroxyphenyl) propionic acid; 3HPPA, 3-hydroxy-3-phenylpropionic acid; SAG, salicylic acid glucoside.

In the case of 4HBA formation, we observed deuterated 3-hydroxy-3-(4-hydroxyphenyl) propionic acid, 4HBAld and 4HBA (Fig. 2A; Table 1), suggesting that the non-oxidative routes also participated in the 4HBA biosynthesis. Recently, it has been reported that vanillin and its glucoside are synthesized by a single enzyme, vanillin synthase, through the two-carbon cleavage of ferulic acid and its glucoside in vanilla pods in a coupled non-oxidative hydratase/lyase reaction^34^. Interestingly, we detected deuterated VA in the early samples from both dsOW62/76 and ZH17 plants (Table 1) but not for deuterated vanillin even at 48 h post feeding treatment, although VA and vanillin pools were at similar levels (Supplementary Table S8). The presence of low levels of 3-hydroxy-(3-methoxy-4-hydroxyphenyl) propionic acid (3H3Me4HPPA) without deuterium labeling implied that vanillin could be synthesized through non-oxidation route at quite a low turnover rate; however, VA might be formed by β-oxidative pathway from feruloyl CoA (Fer-CoA) ester in rice.

### Down-regulation of flavonoid biosynthetic pathway in dsOW62/76 plants

**Figure 3:**
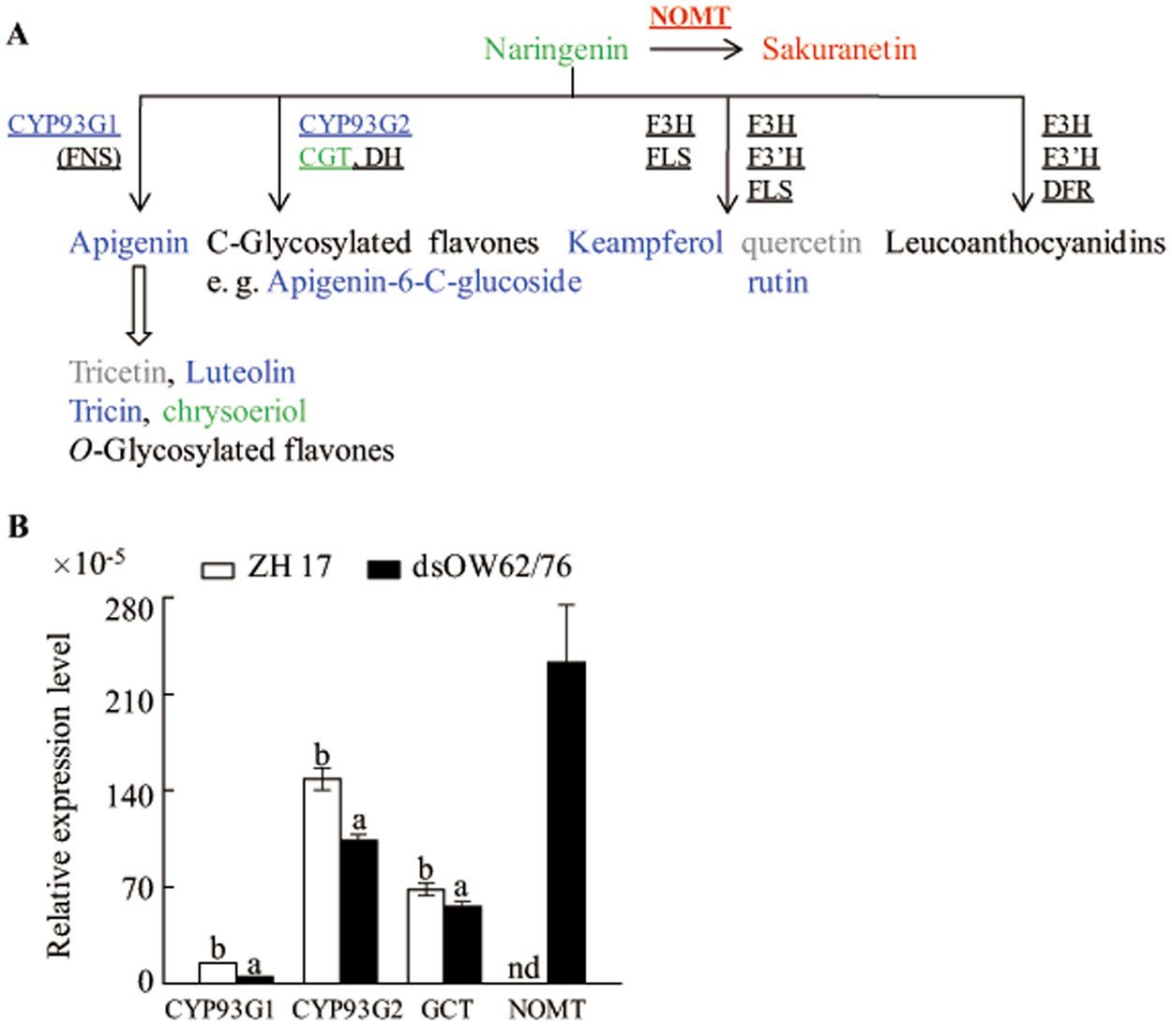
Changes in the flavonoid biosynthesis (**A**) and expression of the related genes (**B**) in dsOW62/76 and ZH17 plants. Naringenin is a common substrate for flavonoid biosynthesis. The font colors and shapes represent the same meanings as described in Fig. 1. A rice *ubiquitin* gene was used as the internal standard for qPCR analysis. Experiments were performed twice with similar results. Values marked with different letters indicate statistically significant differences as analyzed by SAS software (Duncan’s multiple range test, α = 0.05). nd for not detected. Abbreviations: CGT, *C*-glucosyltransferase; CYP93G1, P450 flavone synthase; CYP93G2, P450 flavanone 2-hydroxylase; DH, dehydratase; DRF dihydroflavonol 4-reductase; F3H, flavanone 3-hydroxylase; F3′H, flavanoid 3′-hydroxylase; FLS, flavonol synthase; FNS, flavone synthase; NOMT, naringenin 7-*O*-methylase.

Naringenin, the common precursor for a large number of downstream flavonoids, is synthesized from coumaroyl-CoA (Cou-CoA) together with three moieties of malonyl-CoA catalyzed sequentially by chalcone synthase (CHS) and chalcone isomerase (CHI). There are two kinds of flavone synthases (FNS) that catalyze the conversion of flavanones to flavones. In rice, CYP93G1 functions as an FNSI that converts flavanone naringenin and eriodictyol directly to flavone apigenin and luteolin, respectively, and that they can convert to flavone *O*-glycosides^35^. The biosynthetic route of flavone *C*-glycosides is catalyzed by CYP93G2 (FNSII) with flavanone 2-hydroxylase activities and *C*-glucosyltransferase (GCT) in a way of metabolic channeling^36^. As shown in Supplementary Table S5 and Fig. 3A, most of the flavone and flavonol derivatives accumulate at lower levels in dsOW62/76 comparing with ZH17 plants and levels of transcripts of *CYP93G1*, *CYP93G2* and *GCT* genes are down-regulated in dsOW62/76 (Fig. 3B), suggesting a coordinated control of gene-metabolite pairs. However, the accumulation of phytoalexin sakuranetin, formed by *O*-methylation of naringenin at the 7 position, was elevated in dsOW62/76, although the pool of free naringenin was even marginally lower than that of ZH17 plants. In addition, we also observed an increase in transcript levels of *NOMT* gene (Fig. 3B; Supplementary Table S6), the 7-*O*-methylase gene of naringenin. These findings indicate that the antimicrobial compounds are priorly synthesized in dsOW62/76 plants.

### Massive accumulation of phenolamides in dsOW62/76 plants

**Figure 4:**
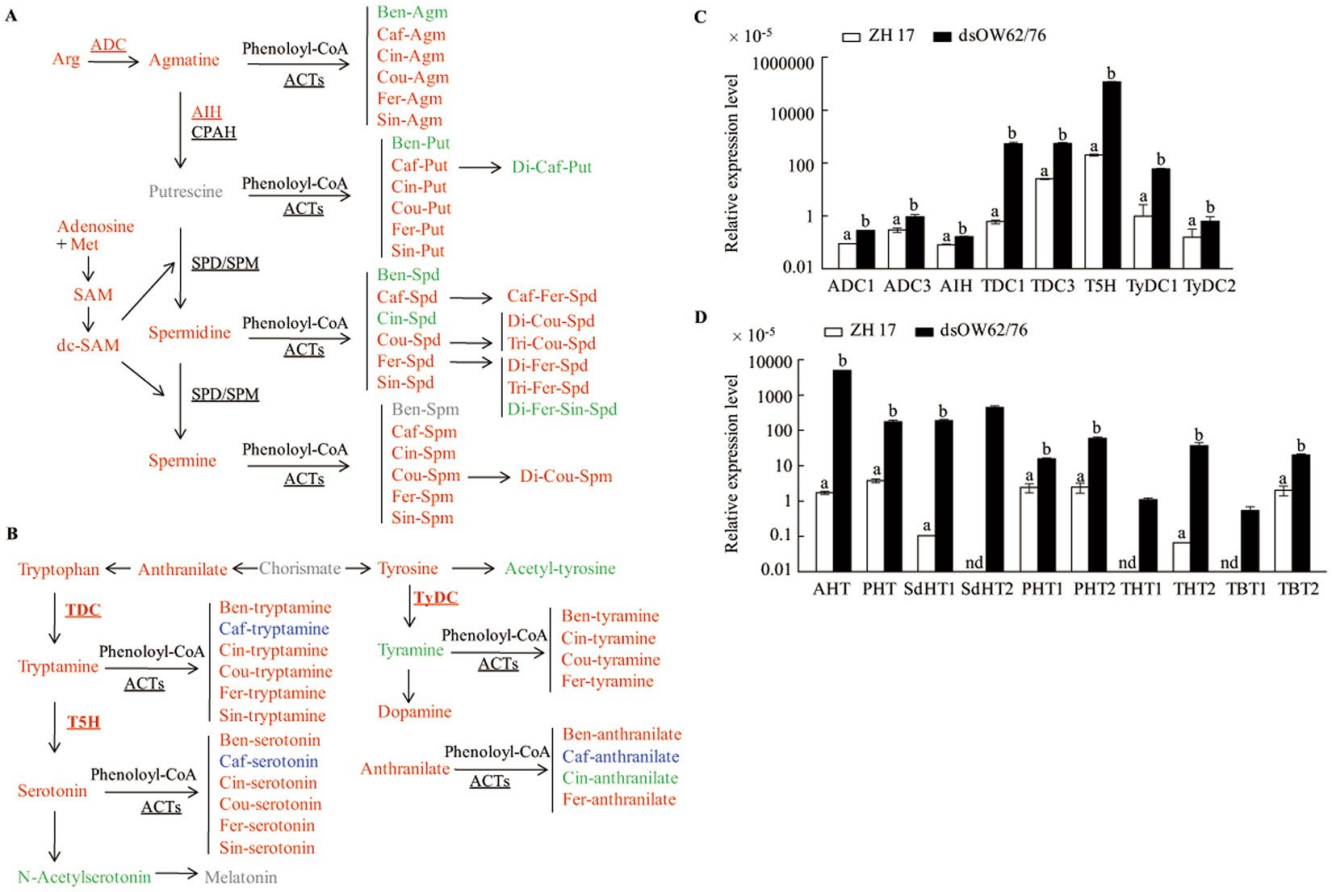
Enhanced accumulation of phenolamides derived from aliphatic (**A**) and aryl (**B**) amines and upregulation of the related genes (**C**,**D**) in dsOW62/76 plants. (**A**) Polyamines (PAs) and agmatine can be conjugated with phenoloyl-CoA to form phenolamides. Similarly, the arylamines derived from chorismate can be converted to phenolamides (**B**). The font colors and shapes represent the same meanings as described in Fig. 1. Transcription levels of the genes for amine generation (**C**) and for phenolamide formation (**D**) were analyzed. Data are means ± SE of three replicates. A rice *ubiquitin* gene was used as the internal standard for qPCR analysis. Values marked with different letters indicate statistically significant differences as analyzed by SAS software (Duncan’s multiple range test, α = 0.05). nd for not detected. Abbreviations: Agm, Put, Spd, and Spm for agmatine, putrescine, spermidine, and spermine, respectively; Ben, Caf, Cin, Cou, Fer, and Sin for benzoyl, caffeoyl, cinnamoyl, coumaroyl, feruloyl, and sinapoyl, respectively; dc-SAM decarboxylated S-adenosylmethionine; SAM S-adenosylmethionine; ACTs, *N*-acyltransferases, in which AHT, PHT, SdHT, and THT for Agm, Put, Spd, and tryptamine hydroxycinnamoyl transferases, respectively and TBT for tryptamine benzoyl transferase; ADC, arginine decarboxylase; AIH, agmatine iminohydolase; CPAH, N-carbamoylputrescine amidohydrolase; SPD/SPM, spermidine/spermine synthase; T5H, trytamine 5-hydroxylase; TDC, tryptophan decarboxylase; TyDC, tyrosine decarboxylase.

Phenolamides are formed by condensation of phenolic acids with amines such as polyamines (PAs) and arylamines. Arg decarboxylase (ADC) enzyme catalyzes the conversion of Arg to agmatine (Agm), which is metabolized to putrescine (Put) sequentially by Agm iminohydrolase (AIH) and *N*-carbamoylputrescine amidohydrolase (CPAH). Put is converted to spermidine (Spd) by the addition of an aminopropyl moiety from decarboxylated *S*-adenosylmethionine (dc-SAM) and to spermine (Spm) by adding two aminopropyl groups at the opposite ends of Put. Levels of these compounds increased in dsOW62/76 plants (Fig. 4A; Supplementary Table S9). We also examined the potential conjugates of the phenoloyl-CoAs such as caffeoyl (Caf) and sinapoyl (Sin) -CoAs condensed with aliphatic amines of Agm, or PAs and found that most of the these phenolamides accumulated at higher levels in dsOW62/76 compared with ZH17 plants (Fig. 4A; Supplementary Table S9).

Trp and Tyr are converted to tryptamine and tyramine by TDC and Tyr decarboxylase (TyDC) enzymes, respectively. Tryptamine 5-hydroxylase (T5H) catalyzes the conversion of tryptamine to serotonin, which plays an important role in defense responses and may lead to the generation of melatonin^37, 38^. The arylamines of tryptamine, tyramine, anthranilate, and serotonin were found to conjugate with acyl-activated phenolic acids in dsOW62/76 plants (Fig. 4B). The conjugates detected were accumulated at higher levels in dsOW62/76 compared with ZH17 except for Caf-arylamide conjugates, which decreased in the RNAi plants (Fig. 4B). Twenty-seven phenolamides undetectable in ZH17 leaves were clearly observed in dsOW62/76. Compounds of Spd conjugating with tri-coumaroyl, tri-feruloyl as well as with hetero-phenoloyls of Caf and Fer moieties were observed only in dsOW62/76 plants. BAHD *N*-acyltransferases catalyze the formation of phenolamides and several genes were identified by genome-wide association study (GWAS) in rice^39–41^. All of the tested genes, including those responsible for amine donor formation or condensation reactions, were strongly activated in dsOW62/76 plants, which may lead to the accumulation of most of the phenolamides (Fig. 4C,D; Supplementary Table S10).

### Preferential synthesis of defense related terpenoids in dsOW62/76 plants

**Figure 5:**
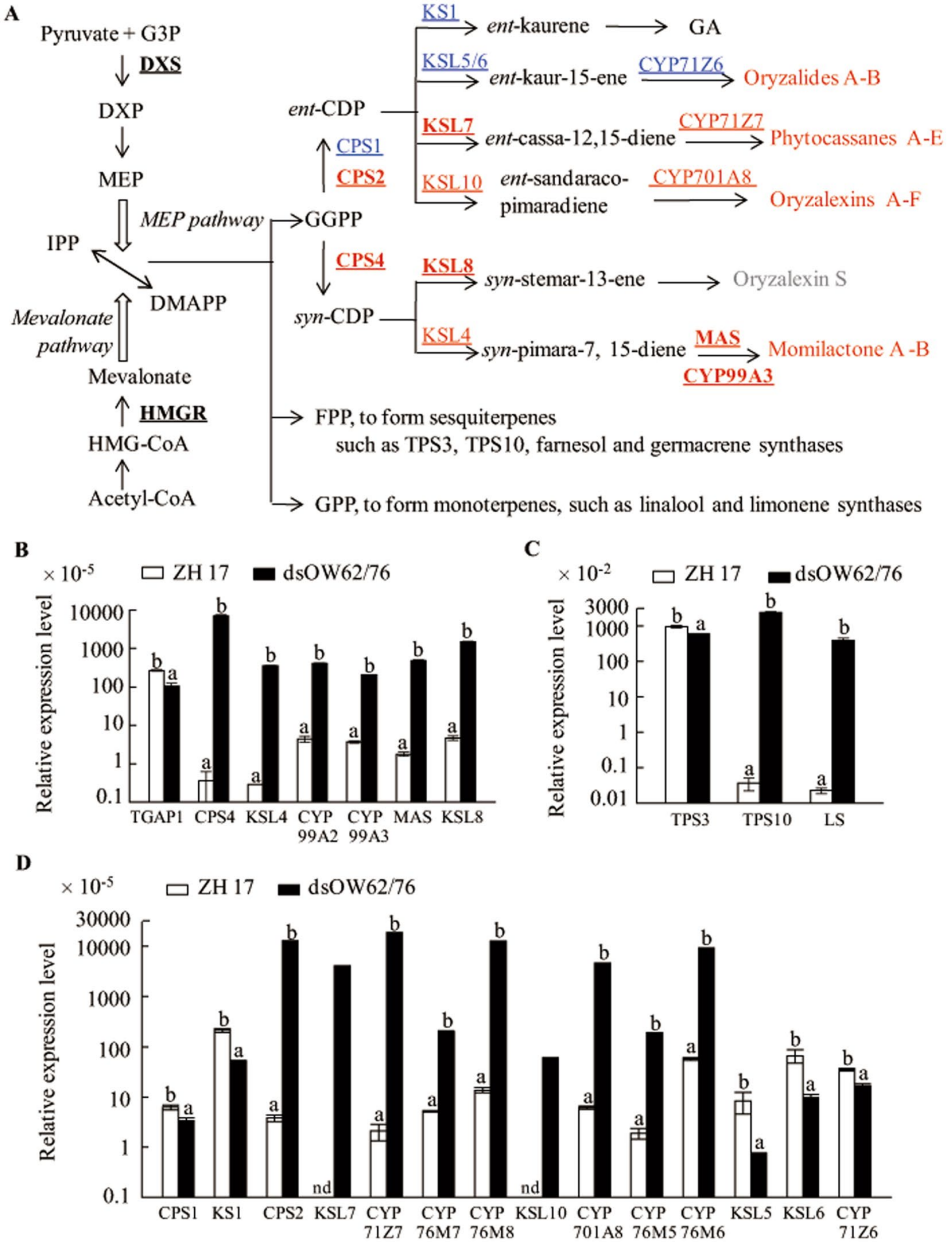
Schematic overview of the changes in terpenoid biosynthesis pathways (**A**) and analysis of the related gene expression (**B**–**D**) in dsOW62/76 and ZH17 plants. (**A**) The cytosolic mevalonate and the plastidial MEP pathways generated IPP and DMAPP are used to produce terpenoids. The font colors and shapes represent the same meanings as described in Fig. 1. (**B**) Expression of the gene cluster related to diterpenoid phytoalexin biosynthesis on chromosome 4, *TGAP1* and *KSL8* genes. (**C**) Expression of terpene synthase genes. (**D**) Expression of the gene cluster related to diterpenoid phytoalexin biosynthesis on chromosome 2, *CPS1*, *KS1*, and *KSL10* genes. Data are means ± SE of three replicates. A rice *ubiquitin* gene was used as the internal standard for qPCR analysis. Values marked with different letters indicate statistically significant differences as analyzed by SAS software (Duncan’s multiple range test, α = 0.05). nd for not detected. Abbreviations: CDP, copalyl diphosphate; DMAPP, dimethylallyl diphosphate; DXP, 1-deoxy-D-xylulose 5-phosphate; FPP, farnesyl diphosphate; GA, gibberellin; GGPP, geranylgeranyl diphosphate; GPP, geranyl diphosphate; HMG-CoA, 3-hydroxy-3-methylglutaryl-CoA; IPP, isopentenyl diphosphate; MEP, 2-*C*-methyl-*D*-erythritol-4-phosphate; CPS, copalyl diphosphate synthase; DXS, DXP synthase; HMGR, 3-hydroxy-3-methylglutaryl-CoA reductase; KS, kaurene synthase; KSL, KS like; LS, linalool synthase; MAS, a dehydrogenase; TPS, terpene synthase.

Two gene clusters have been shown to condition the biosynthesis of diterpenoid phytoalexins in rice. The one on chromosome 4 mediates the formation of momilactones and contains five genes encoding copalyl diphosphate (CDP) synthases 4 (CPS4), kaurene synthase (KS)-like 4 (KSL4), a dehydrogenase MAS, and two CYPs^42, 43^. The other gene cluster on chromosome 2 is responsible for the production of phytocassanes and oryzalides, which encodes enzymes including CPS2, KSL5–7 and seven CYPs from the CYP71 and CYP76 families. Transcription levels of these two gene clusters were dramatically elevated in dsOW62/76 plants except for *KSL5*, *KSL6*, and *CYP71Z6*, which lead to the production of oryzalides (Fig. 5B; Supplementary Table S10). *KSL8* and *KSL10* related to diterpenoid phytoalexin biosynthesis were also upregulated in dsOW62/76, whereas expression levels of *CPS1* and *KS1*, two genes involved in GA biosynthesis, were down-regulated (Fig. 5A,D).

The volatile monoterpenes and sesquiterpenes are also players of plant defense against pathogens and herbivore. Several genes have been functionally characterized to be terpene synthases in rice. OsTPS3 (*Os08g04500*) catalyzes the formation of (*E*)-β-caryophyllene, which is an active signal in attracting parasitic wasps^44, 45^. OsTPS10 (*LOC_Os08g07100*) produces sesquiphellandrene and (*E*)-β-farnesene^45, 46^. Transcript levels of *OsTPS10* were elevated dramatically in dsOW62/76 plants, whereas *OsTPS3*, with high basal levels of expression, was slightly downregulated (Fig. 5C). Overexpression of monoterpene linalool synthase gene (*LS*, *LOC_Os02g02930*) or treatment of rice with linalool enhanced resistance against bacterial pathogen *Xoo* ^47^. Transcript levels of *LS* gene increased by over 1,000 fold in dsOW62/76 plants (Fig. 5C; Supplementary Table S10).

### Increased accumulation of both SA and JA in OsWRKY62 and OsWRKY76 knockout and dsOW62/76 plants

**Figure 6:**
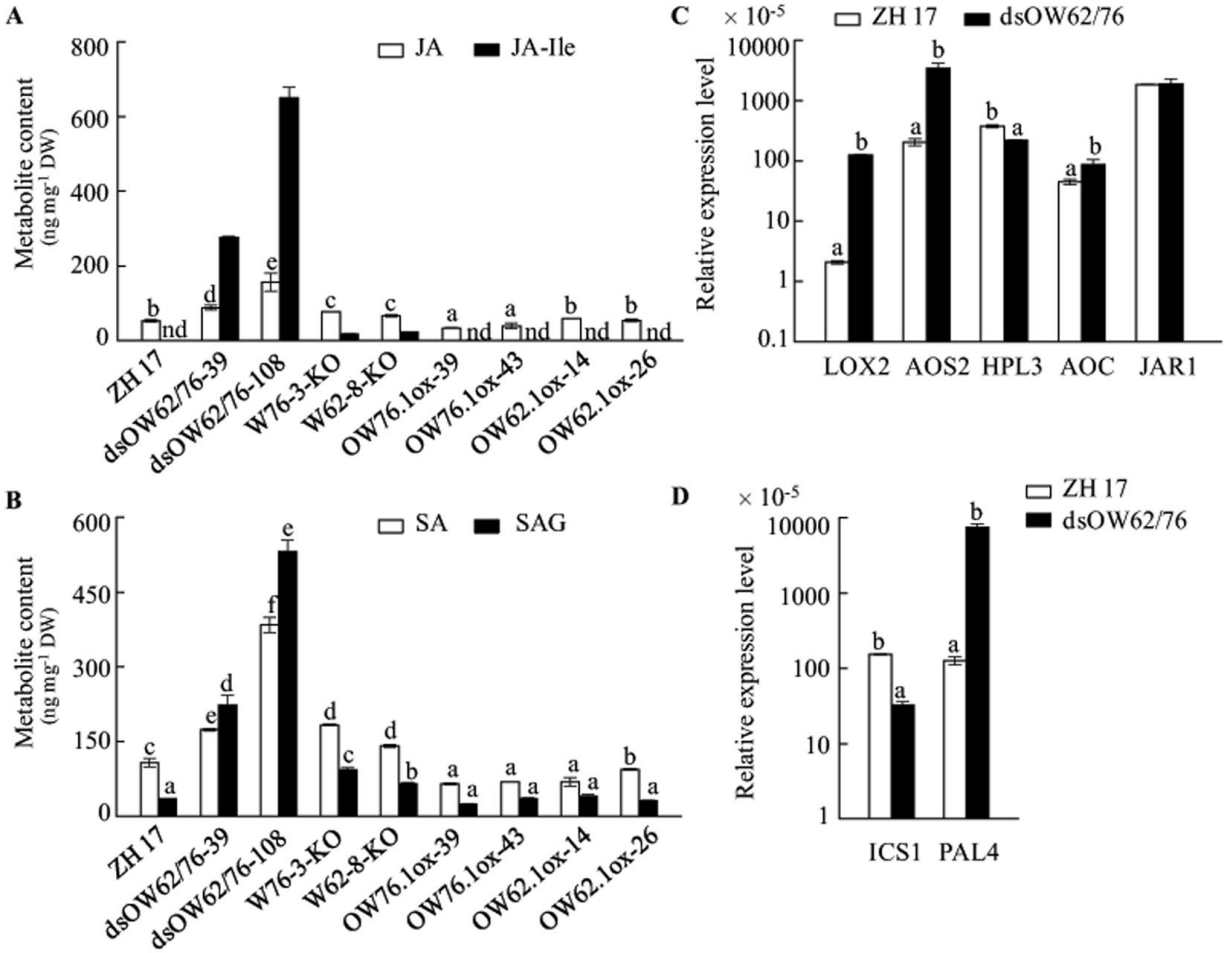
Enhanced accumulation of phytohormones in dsOW62/76 and knockout *OsWRKY62* and *OsWRKY76* lines. (**A**) JA and JA-Ile contents. (**B**) SA and SAG accumulation. Expression of genes related to JA (**C**) and SA (**D**) synthesis. Data are means ± SE of three replicates. Values marked with different letters indicate statistically significant differences as analyzed by SAS software (Duncan’s multiple range test, α = 0.05). The suffix ox is for *OsWRKY62.1*- and *OsWRKY76.1*-overexpressing plants and the suffix KO is for *OsWRKY62* and *OsWRKY76* knockout lines. nd for not detected. Abbreviations: AOC, allele oxide cyclase; AOS, allele oxide synthase; HPL, hydroperoxide lyase; ICS, isochorismate synthase; JAR, JA-conjugating enzyme; LOX, lipoxygenase; PAL, phenylalanine ammonia-lyase.

Phytohormone SA and JA were determined in the transgenic and wild-type plants. JA and JA-Ile levels were remarkably elevated in dsOW62/76, *OsWRKY62* (W62-8-KO) and *OsWRKY76* (W76-3-KO) knockout plants compared to ZH17 (Fig. 6A). Overexpression of *OsWRKY76.1* decreased the accumulation of JA, whereas JA levels did not change significantly in *OsWRKY62.1*-overexpressing plants. Similarly, SA and SAG accumulated at higher levels in dsOW62/76, W62-8-KO and W76-3-KO than in ZH17 plants, and overexpression of *OsWRKY62.1* or *OsWRKY76.1* suppressed SA accumulation (Fig. 6B).

In the transcriptomic analysis, we found that genes associated with JA biosynthesis and JA signaling were enhanced in dsOW62/76, including those encoding lipoxygenase (LOX), allene oxide synthase (AOS), and JAZs (Supplementary Table S11). Results from the qPCR analysis showed an increase in *OsLOX2*, *OsAOS2*, and *OsAOC* (allene oxide cyclase) mRNA accumulation and downregulation of *hydroperoxide lyase 3* (*HPL3*) gene (Fig. 6C) in dsOW62/76 plants. The *HPL3* knockout mutant (*oshpl3*) plants have shown elevated levels of JA and ROS and enhanced resistance against *Xoo* ^48, 49^. In the SA biosynthetic routes, transcription of isochorismate synthase (*OsICS1*) gene was markedly inhibited in dsOW62/76 (Fig. 6D), suggesting that the SA biosynthesis in plastidial might be suppressed. On the other hand, SA synthesis from phenylpropanoid pathway was likely enhanced due to the increase in BA accumulation (Tables 1; Supplementary Table S5) and expression of genes leading to BA biosynthesis (Figs 2B and 6D).

### Stimulation of SA biosynthesis by MeJA treatment in rice plants

**Figure 7:**
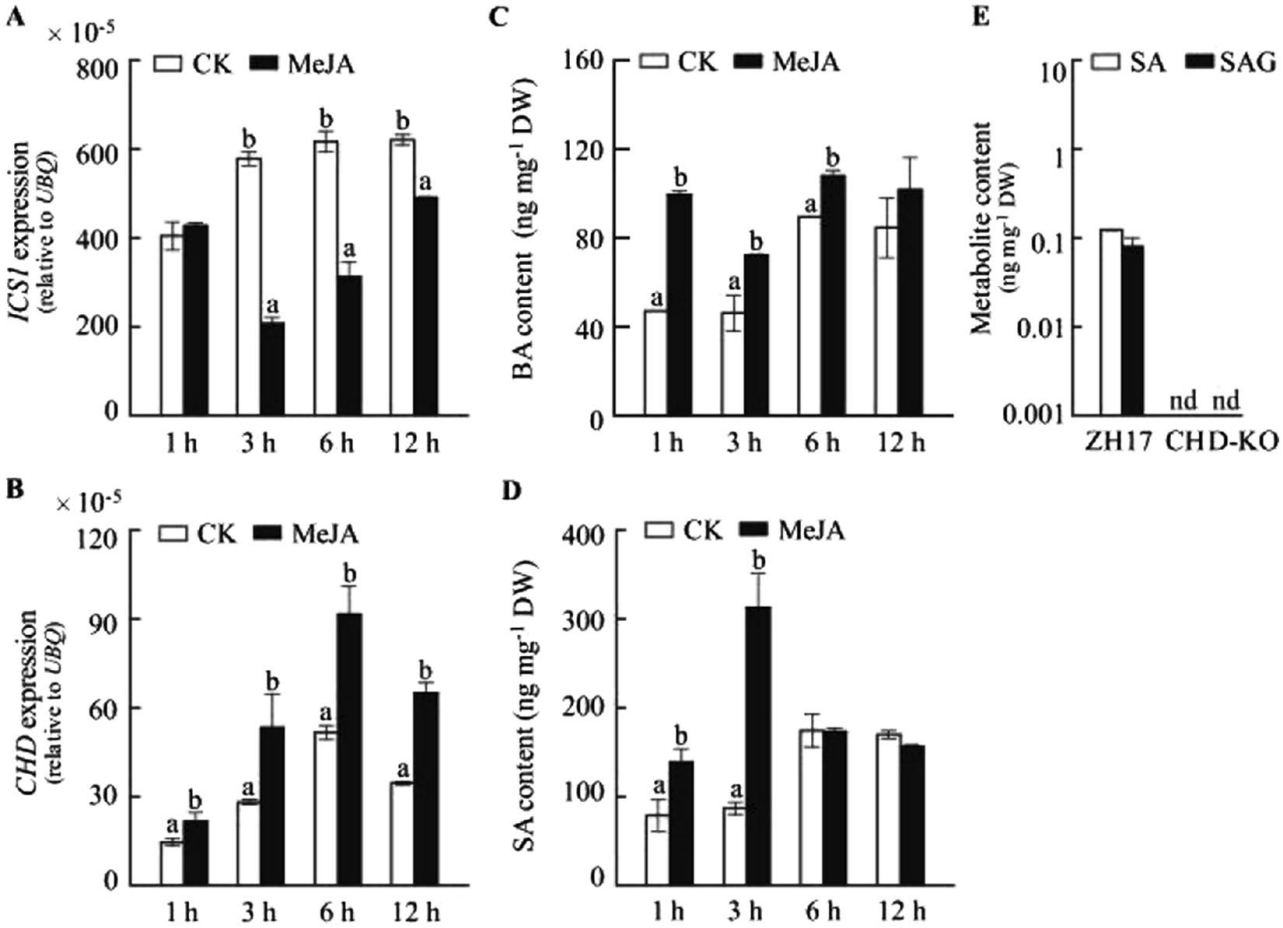
Effect of MeJA on SA biosynthesis and decrease of SA and SAG contents in *OsCHD* mutant. Rice seedlings were treated with MeJA and the leaves were sampled at the designated time points. Transcription level of *ICS1* (isochorismate synthase 1) gene (**A**) and *CHD* (cinnamoyl-CoA hydratase-dehydrogenase) gene (**B**), and BA (**C**) and SA (**D**) levels in the leaves of MeJA treatment. (**E**) SA and SAG contents in *OsCHD* knockout (CHD-KO) calli and the controls (ZH17). Data are means ± SE of three replicates. Values marked with different letters indicate statistically significant differences as analyzed by SAS software (Duncan’s multiple range test, α = 0.05).

To examine the effect of JA on SA biosynthesis, we analyzed expression of genes related to SA biosynthesis and SA accumulation in MeJA treated rice leaves. Transcript levels of *OsICS1* gene were suppressed 3 h post MeJA treatment, whereas that of *OsCHD* gene was elevated (Fig. 7A,B). Following the MeJA treatment, transient but marked increase in BA and SA accumulation was observed in rice leaves (Fig. 7C,D). Further, we measured levels of SA and SAG in *OsCHD* knockout (CHD-KO) calli (Supplementary Fig. S3), and found that the levels of SA and SAG decreased dramatically in CHD-KO compared with the control (Fig. 7E). These observations indicated that the β-oxidation pathway is an important route for SA biosynthesis in rice plant.

## Discussion

Metabolomics, whether used alone or combined with other omics approaches, has been used successfully to understand gene function and mechanisms underlying complex biological processes including development and stress signaling^10, 39, 50, 51^. In this study, we observed massive accumulation of defensive compounds in dsOW62/76 plants. Also, we showed that contents of SA and JA were both elevated in dsOW62/76 and knockout lines of *OsWRKY62* and *OsWRKY76*, suggesting the contribution of *OsWRKY62* and *OsWRKY76* in the regulation of both SA and JA signaling pathways. Further, we showed that β-oxidation route was an important pathway for biosynthetic SA in rice.

During plant-pathogen interactions, ROS plays important roles in the hypersensitive response and can function as a local signal molecule to activate defensive reactions. Auto-accumulation of H_2_O_2_ has been observed in dsOW62/76 plants especially under adverse growth conditions^24^. This study revealed that the oxidative stress-related genes were upregulated in dsOW62/76 plants (Supplementary Tables S2 and S4), in which GSTs, UGTs, and CYPs are involved in detoxification and regulation of the biological activity of metabolites^52^. Metabolite profiling also revealed an imbalance in cellular redox system in dsOW62/76 plants: levels of major reductants of ascorbate and GSH decreased and that of GSSG increased (Supplementary Table S3). Decline of Cys pool in dsOW62/76 potentially confined Cys supply for the synthesis of thiol containing reductants, such as GSH. Maintenance of GSH and ascorbate relies on NAD(P)H content. NADPH, a substrate of NADPH oxidase for the production of superoxide anion as well, can be formed from malate by NADP-malic enzyme, which is localized at *M. oryzae* invasion sites^13^. The accumulation of Arg, Orn, and PA (Supplementary Tables S3 and S9) may imply that H_2_O_2_ could be generated via the actions of diamine and PA oxidases in the process of PA catabolism/interconversion^53^.

Changes in amino acid metabolism have been well documented in response to biotic and abiotic challenges, although their physiological functions and regulatory mechanism under these conditions remain to be fully clarified. Most of the amino acids were found to be more abundant in dsOW62/76 than in ZH17 (Supplementary Table S3). Asp and Glu function as branching points for the synthesis of several amino acids and as donors of the amino group. Levels of Asp and Glu, likely having high free pools, were still increased more than 2-fold in dsOW62/76 plants (Supplementary Table S3). In Arabidopsis, Gln has been demonstrated to play a crucial role in strong resistance against various types of pathogens by enabling nitrogen (N) metabolism to modulate the cellular redox status^54^. Foliage resistance against rice blast fungus was induced by application of Glu in roots^55^. It is interesting to notice the difference in upregulation of *OsWRKY76* and downregulation *OsWRKY62* in the Glu treatment detected by microarray analysis^55^.

Glu synthetase (GS) and glutamine-oxoglutarate aminotransferase (GOGAT) cycle, a major route for the N assimilation in plants, is connected with primary energy metabolism (TCA cycle) and GABA shunt, which bridges the balance between N and C metabolism^56^. Numerous reports showed an increase in GABA levels in response to (a)biotic stresses and here in dsOW62/76 plants than in ZH17 as well (Supplementary Table S3), conferring versatile roles of GABA in connecting metabolism to developmental or environmental cues. In the ABA-deficient *sitiens* mutant of tomato, overactivation of the GABA shunt and the GS/GOGAT cycle to maintain the cell viability in the cells adjacent to the infection sites was important for the resistance against *B. cinerea* ^57^. In the *P. syringae*-Arabidopsis pathosystem, increased levels of GABA at the infection sites, or in GABA transaminase-deficient mutant *pop2-1* enhanced disease resistance^58^. The effector AvrBsT from *X. campestris* pv *vesicatoria* was found to interact with pepper CaADC1 enzyme, catalyzing Agm formation. Further characterization indicated that silencing *CaADC1* in pepper leaves compromised H_2_O_2_, SA, PAs and GABA accumulation and cell death induction^59^.

It has been shown that SA production in plastid requires ICS enzyme to catalyze the formation of isochorismate from chorismate^2^. However, we observed a contrasting effect of dsOW62/76 on the accumulation of free pool of SA and SAG and on the suppression of *OsICS1* transcript levels (Fig. 6B), implying that additional biosynthetic routes may compensate SA production in rice plant. Deuterium labeled BA, SA and even SAG were detected in both dsOW62/76 and ZH17 plants feeding with [^2^H_8_]Phe (Table 1), demonstrating the occurrence of shortening of the C_3_ side chain of *t*-CA. Ratios of isotope-labeled compounds such as BA, SA, SAG, and 2,3-DHBA were at comparative levels, indicating the *de novo* synthesis of SA via BA and similar for SA derivation to maintain SA homeostasis. Using CHD-KO mutant (Fig. 7E), we confirmed that the β-oxidation pathway plays a key role in SA biosynthesis in rice, thereby revealing a major difference of SA biosynthesis between rice and the dicotyledonous model plant Arabidopsis, in which the plastidial ICS route is more important than the phenylpropanoid pathway^60^. The antagonistic effects between SA and JA signaling pathways are well documented. Suppression of *OsICS1* transcription was also observed by MeJA treatment (Fig. 7A); however, *OsCHD* transcript levels were elevated, leading to increase of SA accumulation (Fig. 7B). The results suggest that JA may promote SA accumulation through the dominant SA biosynthetic route in rice plants. This hypothesis was supported partially, at least, by the observations of a simultaneous increase in SA and JA accumulation in rice plants^48, 49^ (Fig. 6A,B).

Activation of the phenylpropanoid pathway is an active defense response of plants that leads to the production of chemicals with antimicrobial activities and/or as precursors of lignin/suberin for the fortification of cell walls. PAL converts *L*-Phe to *t*-CA and releases a molecule of ammonia, which may be reassimilated by the GS enzyme and makes a metabolic link between the phenylpropanoid pathway and the GS/GOGAT cycle^57^. Our data showed that elevated levels of hydroxycinnamic acids in the upper phenylpropanoid pathway were associated with the induction of genes in the pathway in dsOW62/76 plants. For example, transcript levels of *PAL2*, *4* and *7* were increased (Supplementary Table S6), of which *PAL4* has been characterized to be associated with broad spectrum disease resistance in rice^61^. Soluble phenylpropanoids, such as sinapoyl glucose, coniferyl alcohol and coniferin, are important for Arabidopsis against *V. longisporum* ^1^. They have also demonstrated that the phenolics or the precursors of lignin contributed more to defense than lignin *per se*.

It is intriguing that levels of most flavonoids were lower in dsOW62/76 than in ZH17 (Supplementary Table S5), although flavonoids play significant roles in plant disease resistance. Downregulation of flavonoids in dsOW62/76 could be due to significant upregulation of the upper phenylpropanoid pathway, such as an increase in accumulation of phenolic acids and numerous phenolamides, leading to a limited supply of intermediates flux to naringenin. It is partially supported by the observation of marginal decrease naringenin pool in dsOW62/76 (Supplementary Table S5). It is also possible that OsWRKY62 and/or OsWRKY76 directly or indirectly regulate the transcription of genes branching to flavonoids, for example, downregulation of *CYP93G1* and *CYP93G2* genes (Fig. 3B; Supplementary Table S5). Conversely, dsOW62/76 plants on alert of disease resistance were arranged to produce more antimicrobial phytoalexins, such as sakuranetin and phenolamides, especially sakuranetin shared the same precursor naringenin as other flavonoids. It seems also true for an increase in synthesis of terpenoid phytoalexins and downregulation of *CPS1* and *KS1* transcripts, which lead to GA biosynthesis (Fig. 5A,D).

Phenolamides play importance roles in defense or function in growth and developmental processes, but their physiological role remains to be fully evaluated. Several arylamine-conjugated phenolamides have been identified from rice leaves infected with fungal pathogens or treated by UV irradiation^62, 63^. Determination of antimicrobial activities of rice phytoalexins revealed that phenolamides showed inhibitory activities comparable to sukaranetin against rice pathogens^62, 63^. Phenolamides derived from arylmonoamines as well as aryldiamines such as the free tryptamine, serotonin and tyramine are deposited in the cell wall of lesion tissues infected with rice brown spot fungus *Bipolaris oryzae* ^37^, suggested that reinforcement of cell walls is a part of the physical defense system. Peroxidases and laccases are involved in the polymerization and cross-linking to the cell walls. Upregulation of six peroxidase and three laccase genes in dsOW62/76 supported the potential cross-linking of aryl moiety to cell walls (Supplementary Table S6).

It is striking that we identified 56 phenolamides in dsOW62/76 plants and about half of them were not detectable in ZH17 control. The condensation reaction of phenolamide formation is conducted by acyl CoA-dependent BAHD acyltransferases, which also catalyze the addition of an acyl group from the thioester of CoA to oxygen nucleophiles of diverse acceptor molecules^64, 65^. It seems that the formation of Caf-aliphatic amine conjugates is preferred to the Caf-arylamine conjugates in dsOW62/76 (Fig. 4B; Supplementary Table S9), and vice versa, the formation of Ben-arylamine compounds is prior to the Ben-aliphatic amine condensation (Fig. 4A; Supplementary Table S9). It is unclear whether the phenomena are due to the availability of substrates or the specificity of the *N*-acyltransferases, since some BAHD members have wide substrate specificity, such that the products they form *in planta* are probably determined by the relative availability of substrates^41, 64^. Nevertheless, each *N*-acyltransferase member can be knocked out in the dsOW62/76 background to examine its substrate specificity.

Diterpenoid phytoalexins in rice are induced by pathogen infection, JA and UV treatments, and some of the secondary metabolites are secreted into the rhizosphere, functioning as allelochemicals^66^. The assembly of biosynthetic gene clusters is at least beneficent for chromatin modifications and leads to influence access of TFs to the pathway genes. OsTGAP1, a bZIP TF has been shown coordinately in indirect regulation of diterpenoid phytoalexin gene clusters^67^. However, the level of *OsTGAP1* mRNA was declined in dsOW62/76 (Fig. 5B). The data imply that other TFs or OsWRKY62 and OsWRKY76 *per se* are potentially involved in the regulation of the gene clusters. TFs of the same or different family may function in associated ways to regulate gene expression. There are a number of upregulation TF genes, such as *MYB* and *WRKY* family genes in the transcriptomic study (Supplementary Table S2). It will be valuable to clarify the correlation of OsWRKY62 and OsWRKY76 with other WRKY or MYB TF, since the two TF families play important roles in regulating metabolite biosynthesis^14, 15^. Taken together, this study revealed that formation of secondary metabolites is remarkably activated in favor of host defense reactions in dsOW62/76 plants. Combined with previous studies^24, 68^, OsWRKY62 and OsWRKY76 function as negative regulators of disease resistance in rice. The availability of mutants with knockout of *OsWRKY62* and *OsWRKY76* is important for further dissection of the regulatory roles of individual transcription factor or even each of their transcripts.

## Methods

### Plant growth and treatments

The transgenic plants were obtained as described previously^24^. Rice (*Oryza sativa*) seeds were germinated and transplanted in soil containing peat moss and vermiculite (2:1, v/v). Rice plants were grown for three weeks in a growth room under a 14 h light/10 h dark photoperiod at 28 °C. The upper most expanded leaves were harvested from 14 plants at 10:00 am and aliquoted for transcriptomic and metabolomic analyses. Three separated biological replicates were performed.

For isotope feeding, the transgenic dsOW62/76 and wild-type ZH17 seeds germinated were grown in one-half-strength Murashige and Skoog liquid medium. Twelve-day-old seedlings were transferred into 50-ml polypropylene conical tubes in 10 mM MES buffer (pH 5.5) for 12 h starvation treatment and then adding [^2^H_8_]Phe to 5 mM. The upper most expanded leaves were collected at 12 and 48 h after the treatments and lyophilized for compound determination.

For MeJA treatment, leaves of the twelve-day-old seedlings were sprayed with 100 μM MeJA in 10 mM MES buffer (pH 5.5) and the roots were submerged in 10 μM MeJA MES solution. The control plants were treated only with MES buffer containing the same amount of DMSO (0.1%) used for dissolving MeJA. The leaves were sampled at designated time points for gene expression and chemical analysis.

### Determination of metabolites

Preparation of samples was basically the same as described previously^24^. For phytohormone determination, extracts were passed through polymeric reversed phase based solid-phase extraction columns. The eluates were dried by nitrogen gas and dissolved in 50 μl of 90% ethanol in water containing 0.1% formic acid, and were subjected to liquid chromatography-tandem mass spectrometry (LC-MS) analysis with Agilent 6520B quadropole time-of-flight mass spectrometry (Q-TOF MS/MS) equipped with a dual ESI electrospray ion source. Compounds were separated on an Agilent 1260 LC in a Phenomenex LC column (C_18_, 150 mm × 2.1 mm, 3.0 μm) with a gradient elution of 0.05% acetic acid (eluent A) and acetonitrile containing 0.05% acetic acid (eluent B). The elution conditions were 5% B at a flow rate of 0.25 ml min^−1^ for 2 min, consecutive linear gradients to 25% in 8 min, then to 70% in 30 min and then the column was washed with 95% B and equilibrated to the initial conditions. For analysis of hydrophilic compounds, Phenomenex Hilic column (Kinetex Hilic 100 A, 150 mm × 2.1 mm, 2.6 μm) was used with separation conditions of 90% B at a flow rate of 0.2 ml min^−1^ for 2 min, to 70% B in 5 min, then to 50% B in 20 min.

The acquisition, review, and alignment of data were performed with softwares of Agilent MassHunter Acquisition 04.0, MassHunter Qualitative 07.0 and Mass Profinder 06.0, respectively. Full scan MS were recorded through a range of 50–1000 m/z with scan rate at 2 spectra/sec. The ESI source was operated with a nubulizer of 40 psi, drying gas was set at the flow rate of 11 L/min and the temperature was 340 °C, and the capillary voltage was 3.2 kV and 3.0 kV in negative and positive ion mode, respectively. For targeted MS/MS analysis, collision voltages were applied with 10 V, 20 V, 40 V or 60 V for reliable fragmentations.

### MS data processing and compound annotation

Untargeted data acquisition was performed using Agilent MassHunter Workstation Data Acquisition. The data was deconvoluted into individual chemical peaks with Agilent MassHunter Profinder B.06.00 using the untargeted data-mining algorithm Recursive Feature Extractor (RFE). The parameter settings were mass-to-charge ratio (*m/z*) from 50 to 1000, >5000 counts of the absolute peak height, and a limit assigned charge states to a maximum of 1. Lidocaine (0.05 ng) and [^2^H_6_]ABA (10 ng) were used as the inner standards in positive and negative modes, respectively. [^2^H_8_]Phe (50 ng) was used as the inner standard for the analysis of hydrophilic compounds. For metabolites content calculation, peak areas were divided by the area of the internal standard, multiplied by the internal standard weight, and divided by the sample weight. For statistical analysis, the minimum value detected was set as the missing value of a given metabolite, which was not detected. Statistical significance was determined by ANOVA, followed by Duncan test at 5% probability. For normalization, the values were log2 transformed. The hierarchical clusters were obtained using the agglomeration method of average linkage based on the peaks detected. Heatmap was constructed using gene cluster 3.0 and visualized with java treeview. All the data were obtained from at least three replicates.

Metabolites were annotated based on the comparison of ion fragments with the information obtained from literature and database of Metlin (http://metlin.scripps.edu/index.php) and Kegg (http://www.genome.jp/kegg/compound/), or with standard authentic compounds. The metabolites were categorized into four groups accordingly: “verified” confirmed by using authentic compounds; “annotated” characterized by MS and MS/MS ions matching with the information in database or literature; “deduced” determined by specific MS and MS/MS ions; and the “unknown” group do not have any reference.

### RNA-Seq analysis and quantitative real-time PCR

Total RNAs were extracted from the leaves of ZH17 and dsOW62/76 with TRIzol reagent and solexa/illumina sequencing was carried out by Novogene, Beijing, China. Three biological replicates were used. For quantification of gene expression levels, HTSeq v0.6.1 was used to count the reads numbers mapped to each gene. The method of FPKM was used for estimating gene expression levels^69^. Differential expression analysis of ZH17 and dsOW62/76 was performed using the DESeq R package (1.18.0). The resulting P-values were adjusted using the Benjamini and Hochberg’s approach for controlling the false discovery rate. Genes with an adjusted P-value < 0.05 found by DESeq were assigned as differentially expressed. Gene Ontology (GO) enrichment analysis of differentially expressed genes was implemented by the GOseq R package, in which gene length bias was corrected. GO terms with corrected P-value less than 0.05 were considered significantly enriched by differentially expressed genes.

Total RNAs were treated with DNase I to remove possible DNA contaminations and followed by first-strand cDNA synthesis. Quantitative real-time PCR (qPCR) was performed with SYBR premix ExTaq, using gene-specific primers listed in Supplementary Table S12. An *ubiquitin* (*UBQ*) gene was used as an endogenous control in a one-step Real-Time PCR system.

## Electronic supplementary material

Supplementary imformation

Dataset 1

Dataset 2
