# Supplementary material for: Metabolic and transcriptional alternations for defense by interfering OsWRKY62 and OsWRKY76 transcriptions in rice
Source: Sci Rep. 2017 May 30;7:2474. doi: 10.1038/s41598-017-02643-x (PMC5449406; doi:10.1038/s41598-017-02643-x)
Supplement: Supplementary file 1 — Supplementary imformation [file 41598_2017_2643_MOESM1_ESM.doc]

**Metabolic and transcriptional alternations for defense by interfering *OsWRKY62* and *OsWRKY76* transcriptions in rice**

Xiaoxing Liang, Xujun Chen, Cheng Li, Jun Fan, and Zejian Guo*

Key Laboratory of Plant Pathology, MOA; Department of Plant Pathology, China Agricultural University, Beijing 100193, China

*Corresponding author. [guozj@cau.edu.cn](mailto:guozj@cau.edu.cn)

**
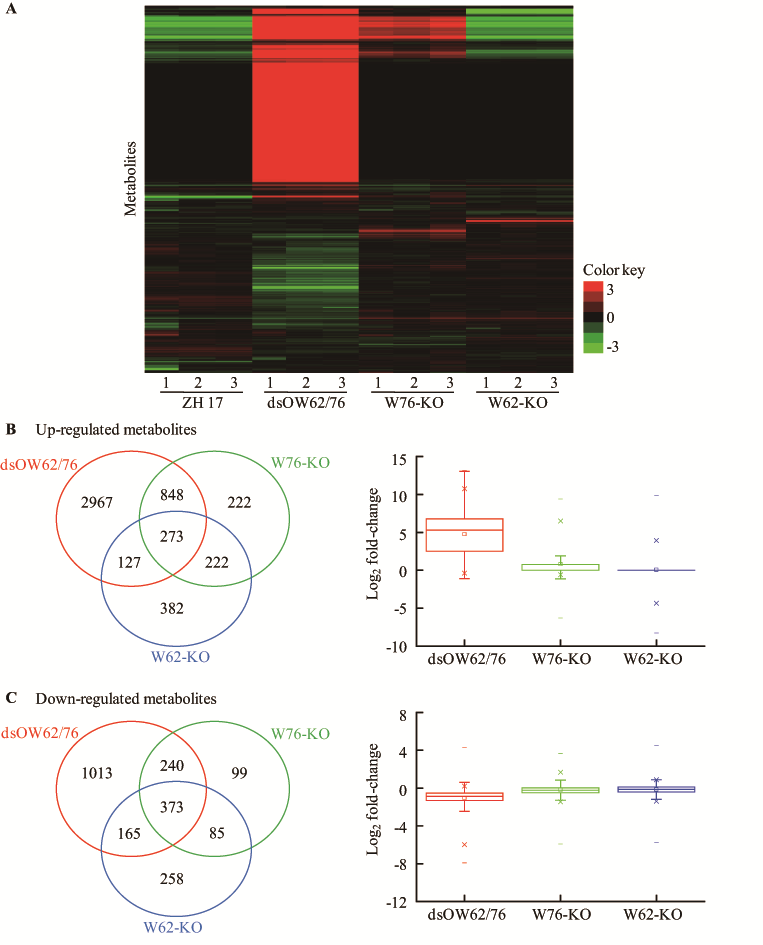
**

**Supplementary Figure S1. Metabolites differances in leaves of *OsWRKY62*, *OsWRKY76* transgenic plant and the control of ZH17. (A)** Heatmap visualization of the metabolite differences among *OsWRKY62* and *OsWRKY76* transgenic plants and the control plants of ZH17. The content value of each metabolite was normalized to average linkage hierarchical clustering. Leaves were collected from three biological experiments of dsOW62/76, W62-KO, W76-KO or from ZH17 as controls, each replicate is visualized in a single column and each of metabolites is represented by a single row. Red indicates high abundance, whereas low relative metabolites are green (color key scale above heatmap). Venn diagram and box plots depicting numbers of up-regulated metabolites **(B)** and down-regulated metabolites **(C)** in dsOW62/76, W62-KO, W76-KO plants compared with the control of ZH17, respectively. dsOW62/76 (red), W76-KO (green), and W62-KO (blue) (T-test, P < 0.05). Box plots show the distribution of log_2_ mutant/ZH17-fold changes of metabolites in dsOW62/76, W62-KO, and W76-KO. The distribution of log_2_ mutant/ZH17-fold changes for up-regulated metabolites (4653 metabolites) and down-regulated metabolites (1979 metabolites) in dsOW62/76, W62-KO, or W76-KO plants.

**
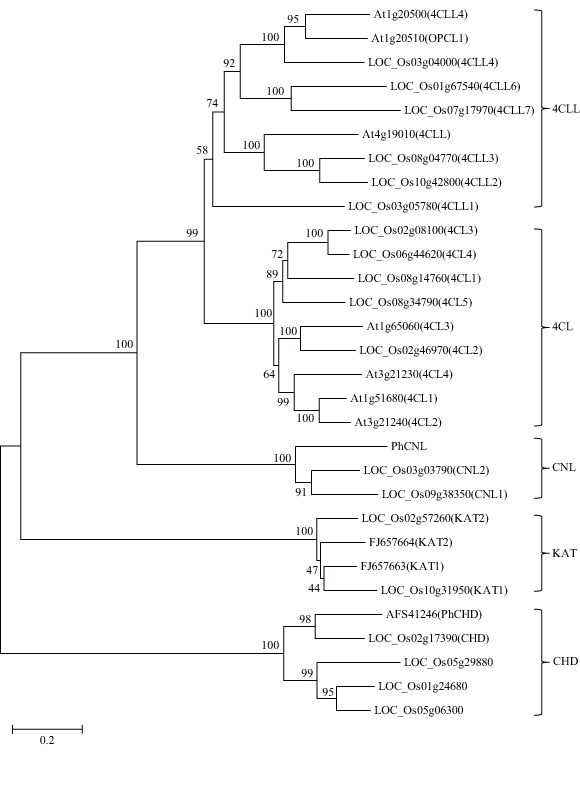
**

**Supplementary Figure S2. Phylogenetic analyses of acyl-activating, KAT and CHD proteins.** An unrooted neighbor-joining cladogram with bootstrap support (1000 replicates) was constructed using the MEGA 6.06. Abbreviations: Os*, Oryza sativa;* At*, Arabidopsis thaliana;* Ph, *Petunia hybrid*; 4CL, 4-coumarate:coenzyme A ligase ; CNL, cinnamate:coenzyme A ligase; CHD, cinnamoyl-CoA hydratase-dehydrogenase; KAT, 3-ketoacyl-CoA thiolase.

**
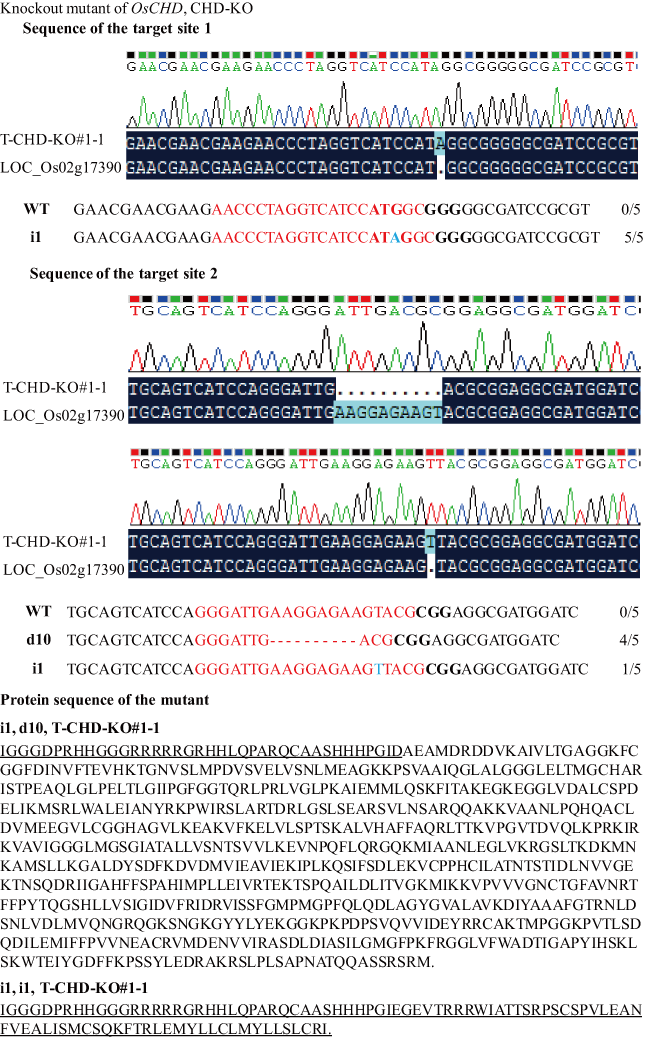
**

**Supplementary Figure S3. Sequence information of *OsCHD* in knockout mutant rice.** The nucleotide sequence of the target site is in red letters and the Pam motif is in black bold. **i1**: one nucleotide insertion is in blue letter; **d10**: 10 nucleotides deletion indicated by dashes. The sequence near the target site was amplified by PCR method and the products were ligated to T-vector for sequencing. The number at the right of the sequence indicates the frequency of the mutant sequences over the total numbers of sequences. The protein sequence alterations resulting from nucleotide sequence mutations are underlined.

**Supplementary Table S3. Compounds varied in the primary metabolism.**

| Pathway | Biochemical Name | ZH17 | dsOW62/76 | Fold change |
| --- | --- | --- | --- | --- |
| TCA cycle | Citrate | 31.64±1.57 a | 131.06±19.08 b | 4.14 |
|  | Fumarate | 9.24±2.27 a | 25.66±0.95 b | 2.77 |
|  | Isocitrate | 62.07±5.54 a | 96.25±12.25 b | 1.55 |
|  | 2-Ketoglutarate | 4.58±0.4 a | 7.11±0.7 b | 1.55 |
|  | Malate | 36.65±6.43 a | 71.65±9.8 b | 1.95 |
|  | Oxaloacetate | 0.11±0.01 | 0.17±0.09 | 1.59 |
|  | Succinate | 2.13±0.26 a | 7.45±1.06 b | 3.51 |
| Amino acid metabolism | Alanine | 61.8±1.62 a | 126.11±4.19 b | 2.04 |
|  | Arginine | 54.63±8.39 a | 151.34±11.6 b | 2.77 |
|  | Asparagine | 21.17±6.16 a | 116.43±26.13 b | 5.50 |
|  | Aspartic acid | 2672.3±91.3 a | 5382.5±94.9 b | 2.01 |
|  | Cysteine | 5.52±0.55 b | 2.39±0.26 a | 0.43 |
|  | Glutamic acid | 1776.62±194.41 a | 4476.57±262.85 b | 2.52 |
|  | Glycine | 0.12±0.01 | 0.09±0.01 | 0.80 |
|  | Glutamine | 58.12±1.6 a | 93.46±8.13 b | 1.61 |
|  | Histidine | 242.14±13.31 a | 842.54±42.89 b | 2.90 |
|  | Isoleucine | 79.15±9.6 a | 121.24±24.07 b | 1.53 |
|  | Leucine | 1662.6±157.1 a | 2057.2±83.5 b | 1.24 |
|  | Lysine | 17.16±3.82 a | 41.33±0.17 b | 2.41 |
|  | Methionine | 2.81±0.24 a | 6.2±0.96 b | 2.20 |
|  | Ornithine | 15.97±1.49 a | 48.12±0.6 b | 3.01 |
|  | Phenylalanine | 28.86±1.94 a | 59.04±3.96 b | 2.05 |
|  | Proline | 19.18±1.07 a | 30.25±2.83 b | 1.58 |
|  | Serine | 14.52±2.25 | 16.16±3.23 | 1.11 |
|  | Threonine | 24.46±4.17 a | 33.4±3.05 b | 1.37 |
|  | Tryptophan | 0.65±0.04 a | 2.67±0.58 b | 4.15 |
|  | Tyrosine | 5.07±1.04 a | 10.82±1.68 b | 2.14 |
|  | Valine | 77.19±8.16 a | 150.01±14.52 b | 1.94 |
| Others | Ascorbate | 17.49±0.29 b | 8.65±0.97 a | 0.49 |
|  | Dehydroascorbate | 5.34±0.39 b | 4.20±0.05 a | 0.79 |
|  | Dopamine | 0.61±0.08 a | 2.19±0.27 b | 3.58 |
|  | γ-Aminobutyric acid | nd | 9.51±2.68 |  |
|  | Glutathione (GSH) | 158.05±17.53 b | 36.26±4.92 a | 0.23 |
|  | GSH oxidized (GSSG) | 1.47±0.36 a | 2.63±0.01 b | 1.8 |
|  | Homoserine | 0.48±0.03 a | 1.09±0.03 b | 2.28 |
|  | Shikimate | 204.27±15.10 a | 334.85±7.60 b | 1.64 |
|  | Pipecolic acid | 11.49±0.58 a | 108.01±3.87 b | 9.41 |
|  | Pyruvate | 378.07±36.42 a | 580.38±6.87 b | 1.54 |
|  | Quinic acid | 4.68±0.77 b | 10.97±1.17 a | 2.34 |
|  | Sucrose | 96.70±7.57 b | 57.64±7.04 a | 0.60 |

nd, not detected. The metabolite contents presented were normalized ng mg^-1^ DW. Data are means ± SE of three replicates. Experiments were repeated two times with similar results. Values marked with different letters indicate statistically significant differences as analyzed by SAS software (Duncan's multiple range test, α = 0.05).

**Supplementary Table S4. Upregulated genes in dsOW62/76 plants by the transcriptomic analysis.**

| Gene ID | Name | Gene Name/Description | ZH17 | dsOW62/76 | Log_2_(dsOW/ZH) | P-value |
| --- | --- | --- | --- | --- | --- | --- |
| LOC_Os03g04410 | ACO | Aconitate hydratase/ Citrate hydro-lyase | 19.72 | 111.54 | 2.50 | 7.05E-03 |
| LOC_Os11g08300 | ALDH3H2 | Aldehyde dehydrogenase | 25.35 | 104.78 | 2.05 | 3.79E-02 |
| LOC_Os04g52440 | GABAT | 4-Aminobutyrate-pyruvate transaminase | 0.08 | 2.22 | 4.78 | 2.73E-02 |
| LOC_Os03g15780 | ASA2 | Anthranilate synthase | 7.98 | 61.15 | 2.94 | 1.88E-04 |
| LOC_Os03g50880 | ASB2 | Anthranilate synthase component II | 6.39 | 35.37 | 2.47 | 7.96E-03 |
| LOC_Os03g18130 | ASN1 | Asparagine synthase | 11.65 | 88.13 | 2.92 | 4.03E-03 |
| LOC_Os01g27750 | DQD-SDH | 3-Dehydroquinate dehydratase / shikimate dehydrogenase | 2.48 | 17.17 | 2.79 | 6.75E-04 |
| LOC_Os09g20820 | ENO | Enolase | 0.30 | 2.89 | 3.28 | 1.19E-03 |
| LOC_Os06g29220 |  | Formate dehydrogenase 2 | 0.24 | 1.98 | 3.07 | 4.59E-03 |
| LOC_Os03g13300 | GAD | Glutamate decarboxylase | 0.26 | 4.52 | 4.10 | 7.47E-06 |
| LOC_Os04g45970 | GDH | Glutamate dehydrogenase | 3.92 | 32.74 | 3.06 | 5.22E-03 |
| LOC_Os09g29200 | GST | Glutathione S-transferase | 151.67 | 858.84 | 2.50 | 1.12E-02 |
| LOC_Os10g38360 | GST | Glutathione S-transferase | 2.42 | 47.41 | 4.29 | 5.78E-05 |
| LOC_Os08g23150 | IGPS | Indole-3-glycerol phosphate synthase | 4.64 | 42.92 | 3.21 | 6.51E-05 |
| LOC_Os09g08130 | IGPS | Indole-3-glycerol phosphate synthase | 0.47 | 21.01 | 5.49 | 2.92E-05 |
| LOC_Os12g24650 | LAP | Leucyl aminopeptidase | 0.04 | 9.81 | 8.01 | 4.91E-02 |
| LOC_Os03g09910 | ALD1 | LL-diaminopimelate aminotransferase | 0.30 | 68.78 | 7.83 | 5.23E-22 |
| LOC_Os09g37540 |  | Lysine decarboxylase-like protein | 7.09 | 148.31 | 4.39 | 2.37E-04 |
| LOC_Os08g33720 | MDH | Malate dehydrogenase | 13.35 | 69.43 | 2.38 | 3.07E-02 |
| LOC_Os01g43460 |  | Malic acid transport protein | 0.92 | 5.07 | 2.46 | 1.09E-02 |
| LOC_Os05g50770 |  | Malic acid transport protein | 0.02 | 1.56 | 6.08 | 3.11E-06 |
| LOC_Os03g48750 | OXO1 | Oxalate oxidase 1 | 0.00 | 0.93 | Inf | 1.66E-03 |
| LOC_Os07g49520 | OGDH | 2-Oxoglutarate dehydrogenase E1 component | 3.65 | 14.77 | 2.02 | 4.50E-02 |
| LOC_Os05g10650 | PFK | 6-Phosphofructokinase 1 | 1.35 | 14.38 | 3.42 | 1.59E-03 |
| LOC_Os04g55720 | PGDH | 3-Phosphoglycerate dehydrogenase | 16.09 | 72.03 | 2.16 | 2.39E-02 |
| LOC_Os02g16630 | TRP | Phosphoribosylanthranilate isomerase | 5.15 | 22.67 | 2.14 | 4.35E-02 |
| LOC_Os06g04280 | EPSPS | 3-Phosphoshikimate 1-carboxyvinyltransferase | 24.30 | 120.04 | 2.30 | 1.73E-02 |
| LOC_Os10g40360 | PDH | Proline dehydrogenase | 7.73 | 44.90 | 2.54 | 5.44E-03 |
| LOC_Os06g12150 | SK | Shikimate kinase | 1.55 | 7.66 | 2.31 | 3.81E-02 |

The values represent means of three replicates. The resulting P-values were adjusted using the Benjamini and Hochberg’s approach for controlling the false discovery rate. dsOW/ZH for the ratio of dsOW62/76 to ZH17.

**Supplementary Table S5. Changes of the compounds derived from phenylpropanoid metabolite pathway.**

| Pathway | Biochemical Name | ZH17 | dsOW62/76 | Fold change |
| --- | --- | --- | --- | --- |
| Benzenoids | *p*-Aminobenzoic acid^#^ | 15.71±0.94 a | 30.41±3.71 b | 1.94 |
|  | Anthranilate^#^ | 4.1±0.43 a | 12.45±0.53 b | 3.04 |
|  | Benzoic acid | 30.31±1.92 a | 98.7±20.51 b | 3.26 |
|  | *p*-Hydroxybenzaldehyde | 0.19±0.01 a | 0.87±0.19 b | 4.46 |
|  | *p*-Hydroxybenzoic acid | 2.55±0.77 a | 27.35±9.36 b | 10.72 |
|  | Vanillic acid | 0.11±0.03 a | 0.22±0.03 b | 1.94 |
|  | Vanillin | 0.13±0.03 a | 0.29±0.04 b | 2.26 |
| Phenylpropanoids | Caffeic acid | 0.07±0.01 a | 0.44±0.05 b | 5.61 |
|  | *t*-Cinnamic acid | nd | 0.37±0.22 |  |
|  | *p*-Coumaric acid | 0.21±0.03 a | 1.16±0.19 b | 5.45 |
|  | Ferulic acid | 0.34±0.01 a | 1.06±0.09 b | 3.12 |
|  | 5-Hydroxyferulic acid | 0.22±0.03 b | 0.15±0.01 a | 0.68 |
|  | Sinapic acid | 0.09±0.01 a | 0.26±0.03 b | 2.84 |
| Flavonoids | Apigenin | 0.57±0.11 | 0.55±0.04 | 0.96 |
|  | Apigenin-6-C-glucoside | 93.99±3.21 b | 15.03±0.62 a | 0.16 |
|  | Apigenin-di-*C*,*C*-pentoside | 24.06±2 | 29.22±0.75 | 1.21 |
|  | Chrysoeriol | 6.06±0.38 | 6.34±0.34 | 1.05 |
|  | Chrysoeriol 5-*O*-hexoside | 11.79±0.48 b | 7.08±0.82 a | 0.60 |
|  | Chrysoeriol 7-*O*-hexoside | 65.33±6.14 b | 26.44±1.51 a | 0.40 |
|  | Kaempferol | 2.01±0.22 b | 1.27±0.02 a | 0.63 |
|  | Kaempferol 3-glucoside | 9.73±0.79 b | 1.98±0.25 a | 0.20 |
|  | Kaempferol 3-rungioside | 997.66±93.3 b | 498.61±40.38 a | 0.50 |
|  | Luteolin | 0.73±0.08 | nd |  |
|  | Luteolin 6-C-glucoside | 199.83±17.19 b | 88.33±0.15 a | 0.44 |
|  | Naringenin | 0.37±0.00 | 0.32±0.03 | 0.86 |
|  | Rutin | 1192.35±97.97 b | 469.2±25.55 a | 0.39 |
|  | Sakuranetin | 0.01±0.01 a | 1.70±0.04 b | 272 |
|  | Seigin | 1.47±0.13 b | 0.86±0.23 a | 0.59 |
|  | Tricin | 444.91±31.49 b | 345.02±4.71 a | 0.78 |
|  | Tricin 4'-glucoside | 71.92±4.85 b | 23.02±0.86 a | 0.32 |
|  | Tricin 5-glucoside | 440.48±30.91 b | 241.53±11.85 a | 0.55 |
|  | Tricin 7-glucoside | 219.51±16.84 | 207.8±1.81 | 0.95 |

^#^Compound derived from the shikimate pathway. nd, not detected. The metabolite contents presented were normalized ng mg^-1^ DW. Data are means ± SE of three replicates. Experiments were performed twice with similar results. Values marked with different letters indicate statistically significant differences as analyzed by SAS software (Duncan's multiple range test, α = 0.05).

**Supplementary Table S6.** Expression of genes in the phenylpropanoid pathway and cell wall formation.

| Gene ID | Name | Gene Name/Description | ZH17 | | dsOW62/76 | | Log_2_(dsOW/ZH) | P-value |
| --- | --- | --- | --- | --- | --- | --- | --- | --- |
| LOC_Os05g06380 |  | Benzyl alcohol benzoyl transferase | | 0.00 | 1.07 | Inf | | 2.44E-03 |
| LOC_Os08g38900 | CCoAMT-2 | Caffeoyl-CoA O-methyltransferase 2 | | 133.46 | 650.77 | 2.29 | | 8.74E-03 |
| LOC_Os06g10210 | CHI | Chalcone isomerase | | 0.84 | 5.43 | 2.70 | | 5.16E-03 |
| LOC_Os07g34260 | CHS | Chalcone synthase 1 | | 0.21 | 417.89 | 10.97 | | 6.72E-04 |
| LOC_Os02g26810 | C4H | Cinnamate 4-monooxygenase | | 0.01 | 0.78 | 7.02 | | 1.61E-04 |
| LOC_Os09g04050 |  | Cinnamoyl-CoA reductase | | 4.36 | 27.98 | 2.68 | | 9.69E-04 |
| LOC_Os08g34280 |  | Cinnamoyl-CoA reductase | | 21.64 | 187.79 | 3.12 | | 2.20E-02 |
| LOC_Os02g09490 | CAD2 | Cinnamyl alcohol dehydrogenase 2 | | 15.63 | 97.01 | 2.63 | | 1.32E-03 |
| LOC_Os08g34790 | 4CL5 | 4-Coumarate--CoA ligase 5 | | 1.23 | 28.20 | 4.52 | | 1.70E-02 |
| LOC_Os01g67540 | 4CLL6 | 4-Coumarate--CoA ligase-like 6 | | 1.79 | 8.99 | 2.32 | | 8.22E-03 |
| LOC_Os10g36848 | F5H | Ferulate-5-hydroxylase | | 1.43 | 26.11 | 4.19 | | 5.71E-04 |
| LOC_Os04g56700 | F3H | Flavanone 3-hydroxylase | | 0.01 | 1.39 | 6.61 | | 4.21E-05 |
| LOC_Os10g39140 | F3H | Flavanone 3-hydroxylase | | 12.03 | 330.69 | 4.78 | | 2.37E-02 |
| LOC_Os10g40934 | F3H | Flavanone 3-hydroxylase | | 2.84 | 19.81 | 2.80 | | 4.57E-03 |
| LOC_Os10g41020 | F3H | Flavanone 3-hydroxylase | | 0.11 | 1.82 | 4.00 | | 1.53E-04 |
| LOC_Os03g03034 | F3H | Flavanone 3-hydroxylase-like protein | | 6.79 | 66.21 | 3.29 | | 7.33E-05 |
| Os10g0536450 | F3H | Flavanone 3-hydroxylase-like protein | | 13.61 | 317.11 | 4.54 | | 1.84E-02 |
| LOC_Os09g17560 | FOMT | Flavonoid 7-O-methyltransferase-like | | 0.39 | 3.28 | 3.08 | | 7.03E-04 |
| LOC_Os05g45110 |  | Flavonol glucosyltransferase | | 0.03 | 1.78 | 6.00 | | 3.50E-02 |
| LOC_Os03g55030 | UGT | Glucosyl transferase | | 21.67 | 6.53 | -1.73 | | 2.69E-02 |
| LOC_Os04g46970 | UGT | Glucosyltransferase | | 0.91 | 20.29 | 4.47 | | 9.41E-10 |
| LOC_Os02g35020 | UGT | Glycosyl transferase | | 0.00 | 1.36 | 8.22 | | 2.08E-08 |
| LOC_Os04g42760 | UGT | Glycosyl transferase | | 0.01 | 4.01 | 8.88 | | 1.47E-07 |
| LOC_Os06g07600 | UGT | Glycosyltransferase | | 1.01 | 5.36 | 2.41 | | 7.25E-03 |
| LOC_Os01g31370 | UGT | Glycosyltransferase | | 0.29 | 4.22 | 3.89 | | 2.60E-05 |
| LOC_Os02g22380 | UGT | Glycosyltransferase | | 14.07 | 60.72 | 2.11 | | 3.35E-02 |
| LOC_Os10g31950 | KAT 1 | 3-Ketoacyl-CoA thiolase 1 | | 23.56 | 174.45 | 2.89 | | 4.74E-02 |
| LOC_Os11g42200 | LAC19 | Laccase-19 | | 0.15 | 19.75 | 7.08 | | 4.71E-04 |
| LOC_Os12g15680 | LAC24 | Laccase-24 | | 0.04 | 50.53 | 10.23 | | 3.82E-27 |
| LOC_Os01g63180 | LAC6 | Laccase-6 | | 1.44 | 25.49 | 4.14 | | 3.86E-03 |
| LOC_Os12g13800 | NOMT | Naringenin 7-O-methyltransferase | | 0.00 | 11.49 | Inf | | 4.62E-02 |
| LOC_Os04g49210 |  | Naringenin, 2-oxoglutarate 3-dioxygenase | | 0.66 | 155.18 | 7.87 | | 9.50E-11 |
| LOC_Os04g55740 | PX | Peroxidase | | 0.00 | 0.48 | Inf | | 2.05E-02 |
| LOC_Os01g73200 | PX | Peroxidase | | 20.48 | 112.19 | 2.45 | | 4.46E-03 |
| LOC_Os11g02100 | PX | Peroxidase | | 0.31 | 2.98 | 3.26 | | 4.84E-03 |
| LOC_Os04g59190 | PX | Peroxidase | | 1.05 | 14.37 | 3.78 | | 4.68E-06 |
| LOC_Os12g02060 | PX | Peroxidase 136 | | 0.04 | 4.38 | 6.82 | | 2.19E-04 |
| LOC_Os01g22352 | PX | Peroxidase 15 | | 0.08 | 1.46 | 4.24 | | 3.31E-03 |
| LOC_Os02g41650 | PAL2 | Phenylalanine ammonia-lyase 2 | | 88.44 | 501.67 | 2.50 | | 1.63E-03 |
| LOC_Os02g41680 | PAL4 | Phenylalanine ammonia-lyase 4 | | 2.91 | 211.55 | 6.18 | | 7.84E-03 |
| LOC_Os05g35290 | PAL7 | Phenylalanine ammonia-lyase 7 | | 2.95 | 73.76 | 4.64 | | 4.68E-05 |
| LOC_Os01g59100 | UGT | UDP-glucose:salicylic acid glucosyltransferase | | 0.73 | 7.16 | 3.29 | | 1.18E-04 |
| LOC_Os11g25454 | UGT | UDP-glucuronosyl/UDP-glucosyltransferase family protein | | 1.66 | 9.22 | 2.47 | | 1.63E-02 |
| LOC_Os01g41430 | UGT | UDP-glucuronosyl/UDP-glucosyltransferase family protein | | 0.59 | 5.50 | 3.23 | | 2.12E-02 |
| LOC_Os06g17020 | UGT | UDP-glycosyltransferase | | 0.69 | 5.18 | 2.90 | | 1.81E-02 |

The values represent means of three replicates. The resulting P-values were adjusted using the Benjamini and Hochberg’s approach for controlling the false discovery rate. dsOW/ZH for the ratio of dsOW62/76 to ZH17.

**Supplementary Table S7. The MS/MS ions of the compounds labeled by deuterium in the [^2^H_8_]Phe feeding experiments.**

| Compounds | Formular | RT | CE | MS/MS |
| --- | --- | --- | --- | --- |
| Benzoic acid | C7H6O2 | 19.73 | 10 | 121.0286, 77.0396 |
| [^2^H_5_]Benzoic acid | C7HD5O2 | 19.73 | 10 | 126.0593, 82.0705 |
| Caffeic acid | C9H8O4 | 13.46 | 10 | 179.0349, 135.0458 |
| [^2^H_5_]Caffeic acid | C9H3D5O4 | 13.53 | 10 | 184.0664, 140.0751 |
| Cinnamic acid | C9H8O2 | 22.63 | 10 | 147.0452, 103.0545 |
| [^2^H_7_]Cinnamic acid | C9HD7O2 | 22.41 | 10 | 154.0892, 110.0971 |
| 2,3-dihydrobenzoic acid | C7H6O4 | 14.95 | 10 | 153.0175, 109.0933 |
| [^2^H_3_]2,3-dihydrobenzoic acid | C7H3D3O4 | 14.88 | 10 | 156.0367, 112.0399 |
| 2,3-DHBA-Glc | C13H16O9 | 10.75 | 10 | 315.0727, 255.2355, 153.0141 |
| [^2^H_3_]2,3-DHBA-Glc | C13H13D3O9 | 10.67 | 10 | 318.0920, 258,0084, 156.0329 |
| Ferulic acid | C10H10O4 | 15.98 | 10 | 193.0506, 178.0259, 149.0597, 134.0363 |
| [^2^H_5_]Ferulic acid | C10H5D5O4 | 15.88 | 10 | 198.0820, 183.0562, 154.0907, 139.0667 |
| *p*-Hydroxybenzaldehyde | C7H6O2 | 14.18 | 10 | 121.0286, 92.0259 |
| [^2^H_5_]*p*-Hydroxybenzaldehyde | C7HD5O2 | 13.94 | 10 | 126.0615, 97.0587 |
| 4-Hydroxybenzoic acid | C7H6O3 | 12.11 | 10 | 137.0244, 93.0342 |
| [^2^H_4_]4-Hydroxybenzoic acid | C7H2D4O3 | 12.01 | 10 | 141.0501, 97.0590 |
| 2-Hydroxycinnamic acid | C9H8O3 | 18.58 | 10 | 163.0401, 119.0494 |
| [^2^H_6_]2-Hydroxycinnamic acid | C9H2D6O3 | 18.58 | 10 | 169.0781, 125.0861 |
| 4-Hydroxycinnamic acid | C9H8O3 | 15.69 | 10 | 163.0401, 119.0494 |
| [^2^H_6_]4-Hydroxycinnamic acid | C9H2D6O3 | 15.58 | 10 | 169.0781, 125.0861 |
| 3H2HPPA | C9H10O4 | 11.9 | 10 | 181.0505, 163.0422, 135.0465 |
| [^2^H_5_]3H2HPPA | C9H5D5O4 | 11.9 | 10 | 186.0813, 168.0848, 135.0409 |
| 3H4HPPA | C9H10O4 | 10.83 | 10 | 181.0505, 163.0412, 135.045 |
| [^2^H_5_]3H4HPPA | C9H5D5O4 | 10.83 | 10 | 186.0813, 168.0848, 135.0409 |
| 4H3Me4HPPA | C10H12O5 | 12.55 | 10 | 195.0650, 167.0700, 137.0600 |
| 3HPPA | C9H10O3 | 16.75 | 10 | 165.0557, 147.0485 |
| [^2^H_6_]3HPPA | C9H4D6O3 | 16.75 | 10 | 171.0934, 153.0832 |
| Naringenin | C15H12O5 | 24.22 | 10 | 271.0612, 151.0023, 119.0492 |
| [^2^H_6_]Naringenin | C15H6D6O5 | 24 | 10 | 277.0982, 151.0014, 124.0823 |
| SAG | C13H16O8 | 11.95 | 10 | 299.0755, 137.0241, 93.0346 |
| [^2^H_4_]SAG | C13H12D4O8 | 11.84 | 10 | 303.0755, 141.0497, 97.0603 |
| Salicylic acid | C7H6O3 | 22.78 | 10 | 137.0244, 93.0341 |
| [^2^H_4_]Salicylic acid | C7H2D4O3 | 22.5 | 10 | 141.0501, 97.0584 |
| Sinapic acid | C11H12O5 | 16.35 | 10 | 223.0612, 208.0350, 193.0112, 164.0465, 149.0231 |
| [^2^H_4_]Sinapic acid | C11H8D4O5 | 16.31 | 10 | 227.0850, 212.0598, 197.0389, 168.0713, 153.0487 |
| Vanillic acid | C8H8O4 | 13.33 | 10 | 167.0437, 152.0099, 123.0445, 108.0210, 91.0186 |
| [^2^H_3_]Vanillic acid | C8H5D3O4 | 13.26 | 10 | 170.0545, 155.0282, 126.0625, 111.0394, 94.0365 |
| Vanillin | C8H8O3 | 15.4 | 10 | 151.0401, 136.0184, 108.0231, 92.0277 |

D for deuterium; RT for retention time (min); CE for collision energy (V). Chemical abbreviations: 2,3-DHBA, 2,3-dihydroxybenzoic acid; 2,3-DHBA-Glc, 2,3-DHBA glucoside; 3H2HPPA, 3-hydroxy-(2-hydroxyphenyl) propionic acid; 3H4HPPA, 3-hydroxy-(4-hydroxyphenyl) propionic acid; 3H3Me4HPPA, 3-hydroxy-(3-methoxy-4-hydroxyphenyl) propionic acid; 3HPPA, 3-hydroxy-3-phenylpropioinc acid; SAG, salicylic acid glucoside.

**Supplementary Table S8. Analysis of the compounds labeled by ^2^H isotope in rice leaves fed with [^2^H_8_]Phe for 48 h.**

| Compounds | ZH17 | | | dsOW62/76 | | |
| --- | --- | --- | --- | --- | --- | --- |
|  | **H** | **D** | **H** | | **D** |  |
| Phenylalanine | 173.01±21.31 | 177.26±22.97 | 152.87±3.39 | | 231.67±4.74 |  |
| Benzoic acid | 65.65±0.2 | 25.94±0.39 | 84.27±0.76 | | 28.12±0.61 |  |
| *t*-Cinnamic acid | 0.46±0.06 | 0.5±0.07 | 0.31±0.01 | | 0.51±0.02 |  |
| 2,3-DHBA | 2.32±0.15 | 0.73±0.09 | 6.99±0.36 | | 3.36±0.21 |  |
| 2,3-DHBA-Glc | 84.65±10.23 | 21.01±2.39 | 421.91±6.59 | | 138.49±3.2 |  |
| 4-hydroxybenzaldehyde | 0.25±0.09 | 0.03±0.02 | 0.3±0.06 | | 0.08±0.01 |  |
| 4-hydroxybenzoic acid | 5.22±0.69 | 0.28±0.04 | 11.18±0.52 | | 2.93±0.15 |  |
| 2-hydroxycinnamic acid | 67.19±5.05 | 2.37±0.23 | 69.58±0.24 | | 2.37±0.03 |  |
| 4-hydroxycinnamic acid | 0.34±0.01 | 0.09±0.02 | 0.56±0.09 | | 0.13±0.01 |  |
| 3H2HPPA | 0.24±0.04 | 0.06±0.01 | 0.17±0.02 | | 0.11±0.01 |  |
| 3H4HPPA | 0.19±0.04 | 0.34±0.05 | 0.36±0.02 | | 0.41±0.02 |  |
| 4H3Me4HPPA | 0.15±0.03 | nd | 0.21±0.01 | | nd |  |
| 3HPPA | 0.14±0.01 | 0.34±0.02 | 0.2±0.03 | | 0.45±0.03 |  |
| SAG | 50.85±7.08 | 2.07±0.28 | 119.83±5.14 | | 31.7±1.1 |  |
| Salicylic acid | 187.5±22.56 | 21.91±3.26 | 326.45±15.2 | | 78.22±2.69 |  |
| Vanillic acid | 0.19±0.01 | 0.04±0.01 | 0.26±0.04 | | 0.01±0.01 |  |
| Vanillin | 0.1±0.01 | nd | 0.18±0.02 | | nd |  |

nd, not detected; **D** for deuterium labeled and **H** for non isotope labeled compounds. The internal standard was [^2^H_6_]ABA (10 ng). Data are mean ± SE of three replicates. The experiments were repeated three times with similar results. The metabolite contents presented were normalized ng mg^-1^ DW. Chemical abbreviations: 2,3-DHBA, 2,3-dihydroxybenzoic acid; 2,3-DHBA-Glc, 2,3-DHBA glucoside; 3H2HPPA, 3-hydroxy-(2-hydroxyphenyl) propionic acid; 3H4HPPA, 3-hydroxy-(4-hydroxyphenyl) propionic acid; 3H3Me4HPPA, 3-hydroxy-(3-methoxy-4-hydroxyphenyl) propionic acid; 3HPPA, 3-hydroxy-3-phenylpropioinc acid; SAG, salicylic acid glucoside.

**Supplementary Table S9. Accumulation of phenolamides and the related compounds in dsOW62/76 and ZH17 plants.**

| Pathway | Biochemical Name | ZH17 | dsOW62/76 | Fold change |
| --- | --- | --- | --- | --- |
| Anthranilate conjugates | *N*-Benzoylanthranilate | nd | 396.91±49.12 |  |
|  | *N*-Caffeoylanthranilate | 0.52±0.06 | nd |  |
|  | *N*-Cinnamoylanthranilate | 0.58±0.06 | 0.62±0.07 | 1.07 |
|  | *N*-Feruloylanthranilate | nd | 1.36±0.11 |  |
| Tryptophan metabolism | *N*-Acetylserotonin | 14.13±1.44 | 16.96±1.17 | 1.2 |
|  | *N*-Benzoylserotonin | nd | 9.88±0.61 |  |
|  | *N*-Caffeoylserotonin | 5.9±0.98 b | 3.78±0.48 a | 0.64 |
|  | *N*-Cinnamoylserotonin | nd | 5.93±0.07 |  |
|  | *N-p*-Coumaroylserotonin | nd | 114.66±13.79 |  |
|  | *N*-Feruloylserotonin | nd | 20.28±4.96 |  |
|  | *N-*Sinapoylserotonin | nd | 0.5±0.04 |  |
|  | *N*-Benzoyltryptamine | nd | 676.98±71.71 |  |
|  | *N*-Caffeoyltryptamine | 1.59±0.07 b | 1.22±0.1 a | 0.77 |
|  | *N*-Cinnamoyltryptamine | nd | 34.55±1.67 |  |
|  | *N-p*-Coumaroyltryptamine | nd | 24.85±1.26 |  |
|  | *N*-Feruloyltryptamine | nd | 0.46±0.02 |  |
|  | Serotonin | 12.04±0.1 a | 659.23±102.59 b | 54.7 |
|  | Tryptamine | nd | 15.19±2.14 |  |
| Tyrosine metabolism | Acetyltyrosine | 13.12±1.11 | 12.35±2.36 | 0.94 |
|  | *N*-Benzoyltyramine | 0.57±0.02 a | 33.86±1.63 b | 60.44 |
|  | *N*-Cinnamoyltyramine | 1.17±0.59 a | 102.39±29.24 b | 86.8 |
|  | *N*-*o*-Coumaroyltyramine | 1.97±0.03 a | 17.95±0.81 b | 9.11 |
|  | *N*-*p*-Coumaroyltyramine | nd | 3.97±0.17 |  |
|  | *N*-Feruloyltyramine | 1.74±0.05 a | 2.35±0.14 b | 1.36 |
|  | Tyramine | 11.42±1.08 b | 8.37±1.07 a | 0.73 |
| Arginine metabolism | Agmatine | nd | 2.89±0.38 |  |
|  | *N*-Benzoylagmatine | 16.86±1.55 | 14.96±0.46 | 0.89 |
|  | *N*-Caffeoylagmatine | nd | 3.07±0.6 |  |
|  | *N-*Cinnamoylagmatine | 0.14±0.03 a | 7.9±0.46 b | 60.15 |
|  | *N-o-*Coumaroylagmatine | nd | 0.72±0.07 |  |
|  | *N-p-*Coumaroylagmatine | nd | 2.5±0.12 |  |
|  | *N*-Feruloylagmatine | 22.66±3.47 a | 841.47±124.59 b | 28.3 |
|  | *N'*-Feruloylagmatine | 13.65±2.26 a | 628.65±112.91 b | 46.06 |
|  | *N*-Feruloylhomoagmatine | 10.62±2.78 a | 81.32±15.14 b | 7.66 |
|  | *N'*-Sinapoylagmatine | 2.52±0.22 a | 5.44±0.26 b | 2.16 |
|  | *N*-Sinapoylagmatine | 4.17±0.02 a | 22.52±0.4 b | 5.39 |
|  | Putrescine | nd | nd |  |
|  | *N*-Benzoylputrescine | 0.38±0.01 | 0.42±0.06 | 1.12 |
|  | *N*-Caffeoylputrescine | 0.26±0.03 a | 0.36±0.02 b | 1.37 |
|  | *N-*Cinnamoylputrescine | nd | 8.37±0.43 |  |
|  | *N-o-*Coumaroylputrescine | 51.87±1.77 a | 90.6±6.1 b | 1.75 |
|  | *N-p-*Coumaroylputrescine | 26.09±0.51 | 26.46±4.31 | 1.01 |
|  | *N*-Feruloylputrescine | 22.21±2.74 a | 390.08±29.31 b | 17.56 |
|  | *N*-Sinapoylputrescine | nd | 36.39±2.49 |  |
|  | *N'*, *N''*-Dicaffeoylputrescine | 2.33±0.24 | 2.51±0.4 | 1.07 |

***Supplementary Table S9*** *Continued.*

| Pathway | Biochemical Name | ZH17 | dsOW62/76 | Fold change |
| --- | --- | --- | --- | --- |
| Arginine metabolism | Spermidine | 9.7±0.77 a | 18.46±1.49 b | 1.9 |
|  | *N*-Benzoylspermidine | 0.14±0.01 | 0.15±0.02 | 1.07 |
|  | *N-*Caffeoylspermidine | nd | 0.9±0.13 |  |
|  | *N*-Cinnamoylspermidine | 0.29±0.09 | 0.43±0.08 | 1.5 |
|  | *N-*Coumaroylspermidine | 0.4±0.03 a | 3.59±0.38 b | 9.14 |
|  | *N-*Feruloylspermidine | 0.84±0.07 a | 5.69±1.13 b | 6.81 |
|  | *N*-Sinapoylspermidine | nd | 1.78±0.23 |  |
|  | *N1*-Caffeoyl-*N10*-feruloylspermidine | nd | 1.37±0.06 |  |
|  | *N'*, *N''*-Di-*p*-coumaroylspermidine | 0.10±0.02 a | 1.68±0.14 b | 16.54 |
|  | *N1,N10*-Di-feruloylspermidine | 0.41±0.03 a | 32.33±1.8 b | 79.99 |
|  | *N1,N5*-Di-(hydroxyferuloyl)-*N10*- sinapoylspermidine | 0.71±0.14 a | 1.08±0.19 b | 1.52 |
|  | *N1,N5,N10*-Tri-*p*-coumaroylspermidine | nd | 3.84±0.34 |  |
|  | *N1,N5,N10*-Triferuloylspermidine | nd | 1.13±0.06 |  |
|  | Spermine | 0.47±0.08 a | 0.79±0.12 b | 1.66 |
|  | *N-*Caffeoylspermine | nd | 7.29±0.84 |  |
|  | *N-*Cinnamoylspermine | nd | 3.59±0.46 |  |
|  | *N-p-*Coumaroylspermine | nd | 58.00±4.19 |  |
|  | *N*-Feruloylspermine | nd | 2.89±0.2 |  |
|  | *N-*Sinapoylspermine | nd | 1.34±0.19 |  |
|  | *N'*, *N''*-Di-*p*-Coumaroylspermine | 0.78±0.04 a | 27.22±3.27 b | 35.11 |
| Others | Adenosine | 118.3±6.97 a | 704.36±25.9 b | 5.95 |
|  | S-adenosylmethionine (SAM) | 2.4±0.24 a | 3.69±0.22 b | 1.54 |
|  | Decarboxylated SAM (dc-SAM) | nd | 15.47±3.8 |  |

nd, not detected; The metabolite contents presented were normalized ng mg^-1^ DW. Data are means ± SE of three replicates. Experiments were performed twice with similar results. Values marked with different letters indicate statistically significant differences as analyzed by SAS software (Duncan's multiple range test, α = 0.05).

**Supplementary Table S10. Expression of genes in phenolamide and terpenoid biosynthesis pathway.**

| Gene ID | Name | Gene Name/Description | ZH17 | dsOW62/76 | Log_2_(dsOW/ZH) | P-value |
| --- | --- | --- | --- | --- | --- | --- |
| LOC_Os06g08640 |  | Hydroxycinnamoyl transferase | 0.00 | 1.29 | Inf | 5.46E-06 |
| LOC_Os06g08610 | PHT2 | Putrescine hydroxycinnamoyl transferase 2 | 0.86 | 5.84 | 2.77 | 2.44E-02 |
| LOC_Os11g42290 | TBT1 | Tryptamine benzoyl transferase 1 | 0.00 | 0.64 | Inf | 1.25E-03 |
| LOC_Os11g42370 | TBT2 | Tryptamine benzoyl transferase 2 | 0.00 | 3.77 | Inf | 1.06E-10 |
| LOC_Os10g23820 | THT2 | Tryptamine hydroxycinnamoyl transferase 2 | 0.03 | 0.74 | 4.69 | 4.43E-02 |
| LOC_Os08g04540 | TDC 1 | Tryptophan decarboxylase 1 | 0.15 | 19.10 | 6.97 | 4.32E-08 |
| LOC_Os12g16720 | T5H | Trytamine 5-hydroxylase | 0.07 | 4.34 | 5.96 | 5.28E-03 |
| LOC_Os10g23900 | TyDC 2 | Tyrosine decarboxylase 2 | 0.00 | 1.42 | Inf | 4.84E-03 |
|  |  |  |  |  |  |  |
| LOC_Os07g09190 | DXS | 1-deoxy-D-xylulose-5-phosphate synthase | 0.56 | 171.54 | 8.25 | 1.63E-03 |
| LOC_Os04g09920 | CYP99A3 | 9-*beta*-pimara-7,15-diene oxidase | 0.26 | 14.08 | 5.77 | 1.59E-02 |
| LOC_Os02g36030 | CYP76M5 | Cytochrome P450 family protein | 0.10 | 6.94 | 6.16 | 7.50E-03 |
| LOC_Os02g36110 | CYP76M7 | Cytochrome P450 family protein | 1.35 | 71.62 | 5.73 | 5.86E-03 |
| LOC_Os02g36070 | CYP76M8 | Cytochrome P450 family protein. | 0.08 | 24.90 | 8.31 | 6.20E-11 |
| LOC_Os02g36140 | KSL7 | *Ent*-cassa-12,15-diene synthase | 0.30 | 72.96 | 7.90 | 1.89E-04 |
| LOC_Os06g39780 |  | *Ent*-cassadiene C11-alpha-hydroxylase 1 | 1.28 | 59.60 | 5.54 | 8.71E-05 |
| LOC_Os02g36210 | CPS2 | *Ent*-copalyl diphosphate synthase | 0.22 | 79.22 | 8.52 | 3.32E-03 |
| LOC_Os04g09900 | CPS4 | *Ent*-copalyl diphosphate synthase | 1.44 | 194.20 | 7.08 | 3.32E-04 |
| LOC_Os06g37300 | CYP701A8 | *Ent*-kaurene oxidase | 2.32 | 226.07 | 6.60 | 1.29E-07 |
| LOC_Os06g37224 | CYP701A9 | *Ent*-kaurene oxidase | 0.00 | 9.78 | Inf | 1.53E-04 |
| LOC_Os02g36285 | KS | *Ent*-kaurene oxidase | 0.06 | 9.23 | 7.19 | 9.54E-03 |
| LOC_Os09g31970 | HMGR | Hydroxymethylglutaryl-CoA reductase | 2.30 | 19.91 | 3.12 | 4.95E-02 |
| LOC_Os05g34180 | IPI | Isopentenyl-diphosphate delta-isomerase | 1.78 | 8.42 | 2.24 | 2.34E-02 |
| LOC_Os11g28530 | KSL8 | Kaurene synthase like | 0.50 | 44.18 | 6.47 | 4.83E-05 |
| LOC_Os02g02930 | LS | Linalool synthase | 0.03 | 46.60 | 10.59 | 1.93E-02 |
| LOC_Os04g10010 | MAS | Momilactone A synthase | 0.14 | 23.63 | 7.43 | 1.85E-04 |
| LOC_Os02g36280 | CYP76M6 | Oryzalexin E synthase | 0.07 | 6.03 | 6.44 | 6.03E-03 |
| LOC_Os04g27340 | TPS20 | Terpene synthase | 0.01 | 0.69 | 6.65 | 1.64E-04 |
| LOC_Os04g27190 | TPS | Terpene synthase | 2.15 | 23.62 | 3.46 | 2.45E-06 |
| LOC_Os07g11790 | TPS7 | Terpene synthase 7 | 0.01 | 27.11 | 11.90 | 7.09E-04 |
| LOC_Os04g27670 | TPS | Terpene synthase family | 0.08 | 25.26 | 8.28 | 8.11E-22 |

The values represent means of three replicates. The resulting P-values were adjusted using the Benjamini and Hochberg’s approach for controlling the false discovery rate. dsOW/ZH for the ratio of dsOW62/76 to ZH17.

**Supplementary Table S11.** Expression of genes in related with JA metabolism and signaling pathways.

| Gene ID | Name | Gene Name/Description | ZH17 | dsOW62/76 | Log_2_(dsOW/ZH) | P-value |
| --- | --- | --- | --- | --- | --- | --- |
| LOC_Os03g12500 | AOS2 | Allene oxide synthase 2 | 6.18 | 135.53 | 4.45 | 5.46E-06 |
| LOC_Os02g12680 | AOS3 | Allene oxide synthase 3 | 0.00 | 1.19 | Inf | 2.35E-04 |
| LOC_Os04g57090 | JMT | Jasmonate O-methyltransferase | 0.03 | 4.29 | 7.24 | 3.40E-04 |
| LOC_Os10g25290 | JAZ1 | Jasmonate ZIM domain-containing protein 1 | 1.62 | 52.64 | 5.02 | 1.56E-02 |
| LOC_Os03g08320 | JAZ2 | Jasmonate ZIM domain-containing protein 2 | 1.77 | 81.91 | 5.54 | 1.63E-03 |
| LOC_Os03g08330 | JAZ4 | Jasmonate ZIM domain-containing protein 4 | 5.29 | 35.69 | 2.75 | 9.77E-06 |
| LOC_Os10g25230 | JAZ13 | Jasmonate ZIM domain-containing protein 13 | 0.09 | 1.53 | 4.08 | 1.04E-02 |
| LOC_Os08g39840 | LOX9 | Lipoxygenase 9 | 8.20 | 86.52 | 3.40 | 1.19E-04 |
| LOC_Os12g37260 | LOX11 | Lipoxygenase 11 | 25.86 | 385.79 | 3.90 | 3.17E-04 |
| LOC_Os08g39850 | LOX8 | Lipoxygenase 8 | 2.45 | 121.79 | 5.64 | 9.76E-13 |
| LOC_Os03g52860 | LOX-L2 | Lipoxygenase L-2 | 0.01 | 12.77 | 10.34 | 2.05E-15 |

The values represent means of three replicates. The resulting P-values were adjusted using the Benjamini and Hochberg’s approach for controlling the false discovery rate. dsOW/ZH for the ratio of dsOW62/76 to ZH17.

**Supplementary Table S12.** Gene-specific primers for qPCR analysis.

| Pimers | Gene ID | Forward (5' to 3') | Reverse (5' to 3') |
| --- | --- | --- | --- |
| ACT | LOC_Os04g56910 | ACTgCTgCTATgTACTCCCTgAAC | TCgTgCAgTCAACgACCAAgAC |
| AOC | LOC_Os03g32314 | TgTTCgTgTACgAgATCAACgAg | CTgTACAgCTTgTTggTgAAggg |
| AOS2 | LOC_Os03g12500 | TgCTTAAgCCggCCATCACTTC | ATATgCgTAggACggAgCTggTTg |
| CGT | LOC_Os06g18010 | ACgATCCCAACCACCTCTTCAC | AACgTgTTgACgAggATgCC |
| CHD | LOC_Os02g17390 | AAgACAATgCCTggTggAAAgC | TgACCCTgCATgCCTCATTAAC |
| 4CL1 | LOC_Os08g14760 | TTCCACATCTACTCgCTCAAC | gCCTCAgTCATTCCATACCC |
| 4CL2 | LOC_Os02g46970 | CCgCTgTTCCACATCTTCT | TCATCCCgTATCCCTgTCC |
| 4CL3 | LOC_Os02g08100 | ggAgACATCggCTTCgTC | ggTgATTTCTgAgCCTTCTg |
| 4CL4 | LOC_Os06g44620 | ATCTACTCgCTCAACTCCgTg | CAgCCTCAgTCATCCCATAC |
| 4CL5 | LOC_Os08g34790 | gTCCCAATgAAggACgATT | gAATTCCAgCAgCCAACTT |
| 4CLL2 | LOC_Os10g42800 | TggAAgCACTCTTTCTTgTACgC | TCTTATACgACgCCACCTgCTTg |
| 4CLL3 | LOC_Os08g04770 | AgTATgTggCCAAgCAggTTgC | gTgACTTgggAATTgCCTCCAC |
| 4CLL4 | LOC_Os03g04000 | TggACCgTACgTCATgAAAggC | TCTggTgTCAgCgTTgACTgTg |
| 4CLL7 | LOC_Os07g17970 | AgCTTgTCCTgCATTCTTTgCC | CCTgCTTCTTCgTgAggATACg |
| CNL1 | LOC_Os09g38350 | ACgTgACTACgTggTAAggATTgC | CTACAAAggTgTgTggTACTgTgg |
| CNL2 | LOC_Os03g03790 | AAgCATggCgTgTgTTTggAAg | TggTCACTgCCCAgCAATACAC |
| CPS1 | LOC_Os02g17780 | AAgAAATTCgACggAggCgTTC | ATATCCCAAgACgCTCCAACCg |
| CPS2 | LOC_Os02g36210 | gCCTCTTCCATgTgCAgCAAAC | TCCATgCTTCCTCCATTCTgAgC |
| CPS4 | LOC_Os04g09900 | TCggAAgAAACACgCgAATggg | TCgTCgTCTCTCTTAgTTCATCCg |
| CYP701A8 | [LOC_Os06g37300](http://www.ricedata.cn/gene/gene_info.aspx?id=LOC_Os06g37300) | TggCTTCCAAACAAgAgCTTCgAC | AATgTAggATgCCCTTgCCTCTC |
| CYP71Z6 | [LOC_Os02g36150](http://www.ricedata.cn/gene/gene_info.aspx?id=LOC_Os02g36150) | TTggCggAACTCATgCggAATC | TCCCAgCAACAgCTTgTCTTACC |
| CYP71Z7 | [LOC_Os02g36190](http://www.ricedata.cn/gene/gene_info.aspx?id=LOC_Os02g36190) | TTggCTgAACTCATgCggTgTC | CAACAgCAgCTTgTCgTACCTC |
| CYP76M5 | LOC_Os02g36030 | ACgTCAgCgAgAggTTCAACAC | AgTTgACgATgACAggCACgAC |
| CYP76M6 | [LOC_Os02g36280](http://www.ricedata.cn/gene/gene_info.aspx?id=LOC_Os02g36280) | ACTgCTAACgTgCTTgCTACCC | CCTAACTAgTgTgTgACgCgACTg |
| CYP76M7 | LOC_Os02g36110 | ACCCAgTggCACgAgAATAATATg | TgCACTgAACCATCTTCATCCAAC |
| CYP76M8 | [LOC_Os02g36070](http://www.ricedata.cn/gene/gene_info.aspx?id=LOC_Os02g36070) | ACgTgTCCgAgAAgTTCAAgACTg | ATTTgATCAggACAggCACAgC |
| CYP93G1 | LOC_Os04g01140 | TTgTTgACAgTCgTCACAgCAC | AggTAgCgTATCCTgTggTTCg |
| CYP93G2 | LOC_Os06g01250 | CgCTTgTgCAgTgCTTTgACTg | AgTAgAAggAAgggAgCggTTg |
| CYP99A2 | LOC_Os04g10160 | TgTCAACgTCCTTCTCAggATCAg | TCCCTCCCgTgAACATATCCAg |
| CYP99A3 | LOC_Os04g09920 | ggTgATTCCCTTgTCAACgTCCTg | TgTCCCTCCCgTgAACATATCCAg |
| DXP | LOC_Os07g09190 | CAAgTTgAggTCCATgTTCTTgCC | TTggTgTCAATCCTgCCTCCTC |
| HMGR | LOC_Os09g31970 | ATgCCgTTggTggTgTATgTgg | TCgTCCggAgTTAgCgACTTTg |
| HPL3 | LOC_Os02g02000 | AgTACgTgTACTggTCCAACgg | CTTggCggCACACTgTTTgTTC |
| ICS1 | LOC_Os09g19734 | TATggTgCTATCCgCTTCgAT | CgAgAACCgAgCTCTCTTCAA |
| JAR1 | LOC_Os05g50890 | TCCTgCATCCCATTgTgTgTgC | TCCgTgTgTTgTACCggAACTgAg |
| KAT1 | LOC_Os10g31950 | CATCCATTgggTgCTACAggTg | CCCgTCgCTTCATTTCgTTgAg |
| KAT2 | LOC_Os02g57260 | AgCTgggTTCTACgACATAggg | ACTTgTCCTTCCCAACCCATAgC |
| KS1 | LOC_Os04g52230 | TgACAgCCAAggCTTTgAgAgg | ACCACCACTgTgATgAACAAgCAg |
| KS4 | LOC_Os04g10060 | gCTgCTgCATTAgTCCACAgATAC | AgCggATACACggTTggTACTTC |
| KSL10 | LOC_Os12g30824 | TTCAAggTCgAgggTTCCAAgg | TTCgTTTgAgTgggAgCCACAg |
| KSL5 | LOC_Os02g36220 | TgATAgAgCAgTTCAACAgAgCAC | ACACCTgCAgCTCCTCTTATgC |
| KSL6 | LOC_Os02g36264 | ggCAgTCgTgAATgATgCTTCC | TTCTCAACACCTgCTgCTCCTC |
| KSL7 | LOC_Os02g36140 | ACggTTACgATgTTTCCTCAgATg | CCCTTgAAgCgAATCACggAAg |
| KSL8 | LOC_Os11g28530 | ATCCAAATTgggTggCCCAgTg | AAgggTATCCACCATgCAgAgC |
| LS | LOC_Os02g02930 | ACACCTgCTgTTCATgCTAggg | TCCTTCTTgTgCCTCATCCTTgg |
| MAS | LOC_Os04g10000 | ACATggACgACgAgACCATTgAg | AgTCCAACgCCCTTTAggTTCg |

***Supplementary Table S12*** *Continued.*

| Pimers | Gene ID | Forward (5' to 3') | Reverse (5' to 3') |
| --- | --- | --- | --- |
| NOMT | LOC_Os12g13800 | ggAAgCgCAATCTTgCTCAAgTg | TCTTCAggATCTTCACgCACTCC |
| PHT | LOC_Os09g37200 | TTTgTgTTgCATgCgACggTTg | AACACgCggAAATACggTTCgg |
| PHT1 | LOC_Os06g08580 | GAGCCTTGCCAAGTTCAAGGATG | AAGTTGGTGTTGAGCTCGAATGG |
| PHT2 | LOC_Os06g08610 | GCCGGACAAGGCAAACCTATTC | AGTTGTATGTCGACCCAATCTTCG |
| SdHT-1 | LOC_Os12g27220 | TAACTgTgAATgCgCgTggAAg | ACAgggCACAgAAgCCCATTAC |
| SdHT-2 | LOC_Os12g27254 | AgCACTTggCTACAgACCTTgC | CATCTCCCACgTgCATTCACAAC |
| TBT1 | LOC_Os11g42290 | GACACTCCATGAGCACCTTCTG | ACGATGAATGCCTCGCTGTC |
| TBT2 | LOC_Os11g42370 | TCGTTGGAGGAACATGCTATCCC | GGATGTTAGCCGTGGAATTATGCG |
| THT1 | LOC_Os10g23310 | AAGTGCTTCCGGCTAAACAACG | ACACGATCCTTGTCGGCTTG |
| THT2 | LOC_Os10g23820 | CCAATCCACACAAACAACCACCTG | TGTGTGTGTGTCAGTGTACTTAGC |
| TGAP1 | LOC_Os04g54474 | ACATCATgTCgggCATgTggAAg | TCCAgCTgCggTCTCAAATTCTTg |
| TPS10 | LOC_Os08g07100 | gCgCgACTACACgAAATgAAgC | ACgTTAAgCTCTTTCCACCATCg |
| TPS3 | LOC_Os08g04500 | TCACCCTgCACTCCATCAATgC | CACTTgTTCCTCggAAACATgAgC |
| UBQ | LOC_Os06g46770 | gTggTggCCAgTAAgTCCTC | ggACACAATgATTAgggATCA |
